# Reduced structural flexibility for an exonuclease deficient DNA polymerase III mutant†

**DOI:** 10.1039/c8cp04112a

**Published:** 2018-10-31

**Authors:** Hailey L. Gahlon, Alice R. Walker, G. Andre´s Cisneros, Meindert H. Lamers, David S. Rueda

**Affiliations:** a Department of Medicine, Molecular Virology, Imperial College London, Du Cane Road, London W12 0NN, UK; b Single Molecule Imaging Group, MRC London Institute for Medical Sciences, Imperial College London, Du Cane Road, London, W12 0NN, UK; c Department of Chemistry, University of North Texas, 1155 Union Circle, Denton, Texas 76203, USA; d MRC Laboratory of Molecular Biology, Francis Crick Avenue, Cambridge Biomedical Campus, Cambridge, CB2 0QH, UK

## Abstract

DNA synthesis, carried out by DNA polymerases, requires balancing speed and accuracy for faithful replication of the genome. High fidelity DNA polymerases contain a 3′–5′ exonuclease domain that can remove misincorporated nucleotides on the 3′ end of the primer strand, a process called proofreading. The *E. coli* replicative polymerase, DNA polymerase III, has spatially separated (~55 Å apart) polymerase and exonuclease subunits. Here, we report on the dynamics of *E. coli* DNA polymerase III proofreading in the presence of its processivity factor, the β_2_-sliding clamp, at varying base pair termini using single-molecule FRET. We find that the binding kinetics do not depend on the base identity at the termini, indicating a tolerance for DNA mismatches. Further, our single-molecule data and MD simulations show two previously unobserved features: (1) DNA Polymerase III is a highly dynamic protein that adopts multiple conformational states while bound to DNA with matched or mismatched ends, and (2) an exonuclease-deficient DNA polymerase III has reduced conformational flexibility. Overall, our single-molecule experiments provide high time-resolution insight into a mechanism that ensures high fidelity DNA replication to maintain genome integrity.

## Introduction

DNA polymerases (DNA Pols) perform DNA replication by inserting nucleotides from the 3′ end of a growing primer strand. The fidelity of this process is important for maintaining genome integrity. DNA replication fidelity is controlled by a variety of factors including nucleotide selection and the ability of a polymerase to perform proofreading.^1^,^2^ During proofreading, DNA Pols can sense misincorporated nucleotides (nt) and transfer the primer strand from the polymerase (Pol) active site to the exonuclease (Exo) active site where the 3′ nt is excised and subsequently returned to the Pol active site. The relationship between fidelity and proofreading is important since aberrations in proofreading can lead to increased mutagenesis and potentially cancer in higher organisms.^3^–^7^

The DNA polymerase III holoenzyme is the replicative DNA polymerase in *E. coli* comprising ten proteins with a total mass of ~10 MDa. The α subunit of the holoenzyme is the replicative DNA polymerase that belongs to the C family of polymerases and performs fast replication (~10^3^ nt s^−1^), with high fidelity (~10^−6^ error rate) and high processivity (~10^5^ insertions per binding event).^8^–^11^ The ε subunit is the 3′–5′ exonuclease that removes misincorporated nt. In turn, the exonuclease binds the accessory protein θ to form the trimeric complex Pol III core (*i.e.*, α–ε–θ).^12^,^13^ The polymerase and exonuclease active sites are separated by ~55 Å.^14^ How these two subunits work together to coordinate proofreading while balancing fast and accurate DNA synthesis remains largely unknown. In addition, which amino acid residues are involved in the dynamic transfer of the primer strand between the Pol and Exo domains during proofreading is largely unknown. Previous structural data has indicated that tyrosine 453 in Pol III may stabilize an Exo-conformation through an aromatic stacking interaction with a nucleobase on the primer strand.^14^

Mechanistic details of polymerase proofreading dynamics remain elusive because of rapid conformational changes that can be hard to detect and quantify with traditional bulk-averaged biochemical approaches. To circumvent this, we have developed a single-molecule Förster resonance energy transfer (smFRET)^15^,^16^ assay to monitor the proofreading conformational dynamics of Pol III core in the presence of its processivity factor, the β_2_-sliding clamp. We tested various primer template termini containing cognate base pairs and mismatches; templates containing G paired opposite a dideoxy C, matched C, A and T (DNA sequences, Table 1). We also tested Pol III core dynamics at the site of a double mismatch (G:AA, DNA sequence, Table 1). Using this smFRET assay, we measured the kinetics and dynamics of DNA Pol III core. The data show that Pol III core-binding rate constants do not change significantly in the presence of matched or mismatched DNA termini. However, the DNA bound Pol III core complex is highly dynamic and samples numerous conformational intermediates along the proofreading pathway. We determined that an exonuclease deficient polymerase mutant has reduced dynamics compared to wild type, linking a key residue, tyrosine 453, in the thumb domain of the polymerase to protein flexibility and proofreading. Lastly, we observed non-equivalent initial binding conformations for matched and mismatched DNA termini, suggesting a functional role for how Pol III binds initially at the 3′ primer terminus. Altogether, our data reveal perturbations in proof-reading dynamics at both the DNA and protein level, most notably regarding structural changes at the DNA terminus and with the Exo-deficient Pol III mutant.

## Results

**Fig. 1:**
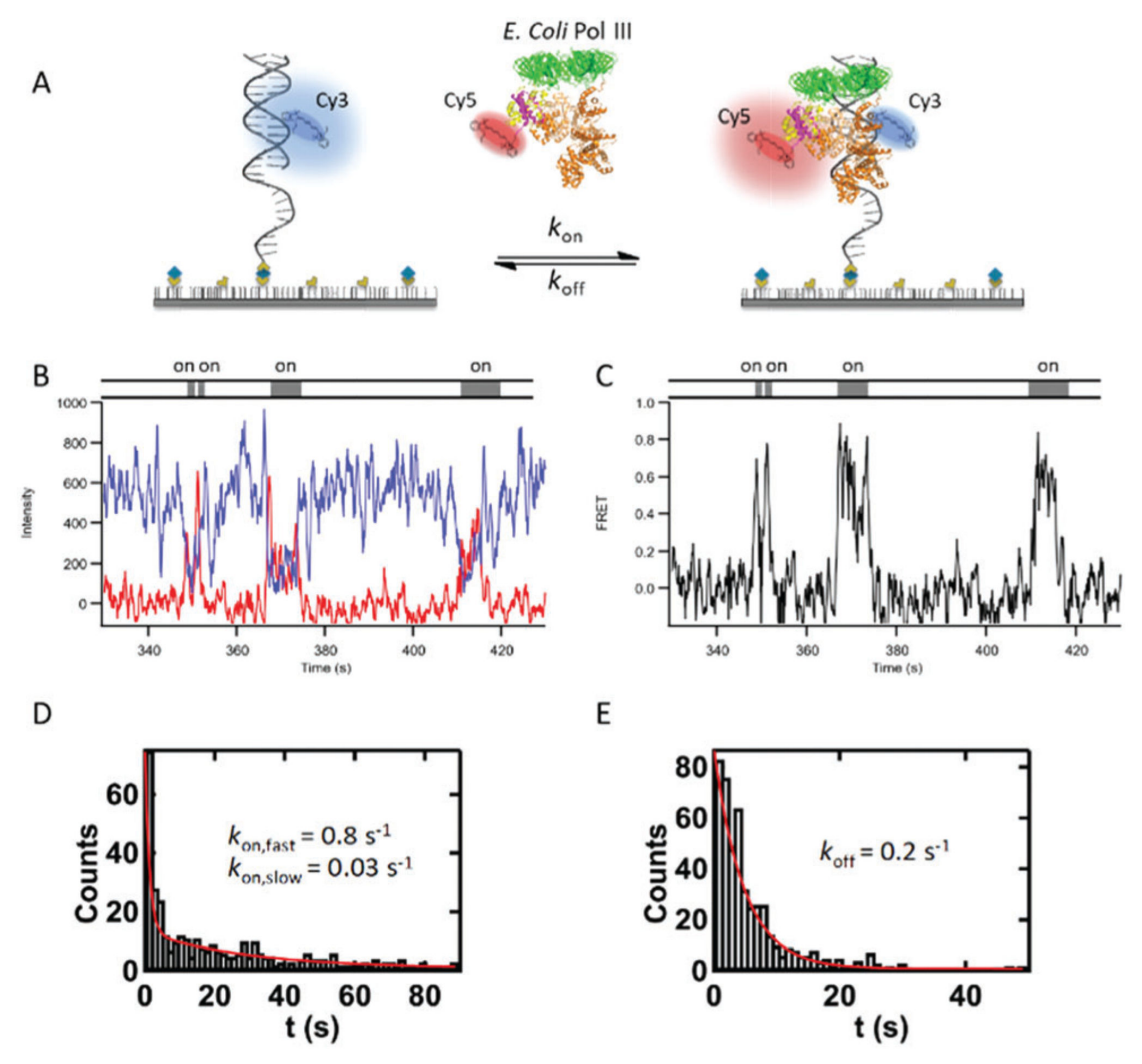
Pol III core binding and dissociation observed by smFRET. (A) Schematic of the smFRET experiment with Cy3 donor emission (blue circle) on DNA and upon protein binding an increase in Cy5 acceptor emission (red circle) *via* fluorescence energy transfer. (B) Representative intensity trajectory with donor intensity in blue and acceptor intensity in red, anti-correlated signals indicate protein binding events. DNA is labeled with a Cy3 donor and contains a terminal G:ddC terminus (see Table 1 for DNA sequence) and Pol III core is labeled with a Cy5 acceptor on the theta subunit containing an E41C mutation to site-specifically label the Cys residue with maleimide chemistry. Single-molecule experiments were performed in 20 mM Hepes, pH 7.6, 5 mM potassium glutamate, 3 mM magnesium acetate, 2 mM DTT, 2 mM Trolox, 25 nM enzyme and 10 μM dCTP. (C) Corresponding FRET trajectory whereby apparent FRET efficiencies were calculated as FRET = *I*_A_/(*I*_D_ + *I*_A_); and *I*_A_ indicates acceptor intensity and *I*_D_ indicates donor intensity and examples of (D) double exponential decay graph for *k*_on_ (pseudo-first order) and (E) single exponential decay graph for *k*_off_.

The smFRET assay enables us to monitor proofreading dynamics and kinetics of Pol III core binding on DNA at matched and mismatched termini (Fig. 1). DNA was labeled with a FRET donor (Cy3) and the Pol III core with an acceptor (Cy5) on the θ subunit to monitor conformational dynamics upon protein–DNA binding (Fig. 1A). Pol III core was incubated with Cy3-labeled DNA on the template strand 7 nucleotides upstream from the 3′ primer terminus in the presence of dCTP, the next correct nucleotide (Table 1). To site-specifically label the protein, we engineered an E41C mutation in the θ subunit (Fig. S1, ESI†). The label did not affect the protein’s polymerase and exonuclease activities (Fig. S2, ESI†). Pol III core alone binds weakly to DNA,^17^,^18^ and relies on the DNA sliding clamp β for processive DNA synthesis. However, the Pol III core affinity for the β-clamp is moderate.^19^ Therefore, to induce long-lasting binding events we used a modified version of the Pol III core that shows ~100-fold improved β binding without affecting DNA synthesis.^18^ This modified complex has been previously shown to be very stable in solution, strongly suggesting that in our smFRET assay the correct Pol III core complex is loaded on to DNA and thus responsible for the FRET signals observed.^18^ In addition, it is important to note that FRET is only observable upon binding of a correctly loaded Pol III core complex. Moreover, in single-molecule PIFE experiments with unlabeled protein, we did not observe binding of Pol IIIα and Pol IIIαε at 25 nM; further demonstrating the observed FRET signal is from the correctly loaded Pol III core complex. Binding of the Pol III core resulted in anti-correlated signals between the Cy3 donor and the Cy5 acceptor fluorophores (Fig. 1B). Apparent FRET efficiencies were plotted over time, indicative of Pol III core binding (Fig. 1B and C). Dwell time analysis yielded pseudofirst order binding and dissociation rate constants, *k*_on_ and *k*_off_, respectively (Fig. 1D and E, Methods). Further, all on rate constants were fit to double exponential decay curves yielding *k*_on,fast_ and *k*_on,slow_ (Fig. 1D), suggesting more than one conformation for Pol III dissociating from its substrate.

### Wild type Pol III core binds matched and mismatched termini with similar rate constants

For wild type Pol III core, short binding and dissociation events were observed with trajectories comprised of multiple binding events. Matched base pairs at the primer–template terminus included G:ddC and G:C (Table 1). For G:ddC, a dideoxycytosine (ddC) was placed at the 3′-end of the primer in order to prevent primer extension by removal of the 3′-hydroxyl group. For the G:C DNA, a non-hydrolyzable dCTP analog was used in the place of dCTP to prevent primer extension. The off rate constants did not vary for either the G:ddC and G:C termini; a *k*_off_ of 0.2 s^−1^ was determined (Table 2 and Fig. S3B, D, ESI†). In addition, the on rate constants did not significantly differ for matched DNA termini and *k*_on,fast_ from 0.6–0.8 s^−1^ and *k*_on,slow_ from 0.02–0.03 s^−1^ was observed (Table 2 and Fig. S3A, C, ESI†).

For mismatched DNA, G:T, G:A and G:AA (Table 1), a phosphorothioate modification was placed at the terminal 3′–5′ phosphate linkage on the primer strand, as this modification prevents exonuclease degradation.^20^,^21^ Therefore, this allows for monitoring proofreading dynamics on mismatches that are not removed in the time scale of our single-molecule experiments. The off rate constants did not vary significantly for either of the mismatched termini; a *k*_off_ of 0.2 s^−1^ was determined for G:T and G:A (Table 2 and Fig. S3F, H, ESI†) and a *k*_off_ of 0.3 s^−1^ for G:AA (Table 2 and Fig. S4B, ESI†). The *k*_on,slow_ did not show appreciable differences amongst the DNA termini, however the *k*_on,fast_ for G:A DNA yielded a rate constant of 0.3 s^−1^, which was 2.5 times slower than for the G:ddC DNA of 0.8 s^−1^ (Table 2).

### Wild type Pol III is dynamic in Pol and Exo modes

FRET efficiency histograms were prepared to determine the degree of dynamics within the population for wild type Pol III binding at varying DNA termini. In general, each histogram is a broad peak exhibiting a ‘single’ population that spans a dynamic FRET range, 0.2–1.0 (Fig. S6–S8, ESI†). For each DNA termini, a similar center of distribution (*X*_0_) was determined, ~0.4 for each DNA. The main difference, however, was for the width of distribution (*σ*). Here, a number of subpopulations exist that could not be individually distinguished. Rather, the histograms reveal an ensemble of conformations that Pol III adopts while binding DNA. In order to investigate conformational differences within these populations at higher resolution, we analyzed dynamic traces with Hidden Markov modeling (HMM).^22^

**Fig. 2:**
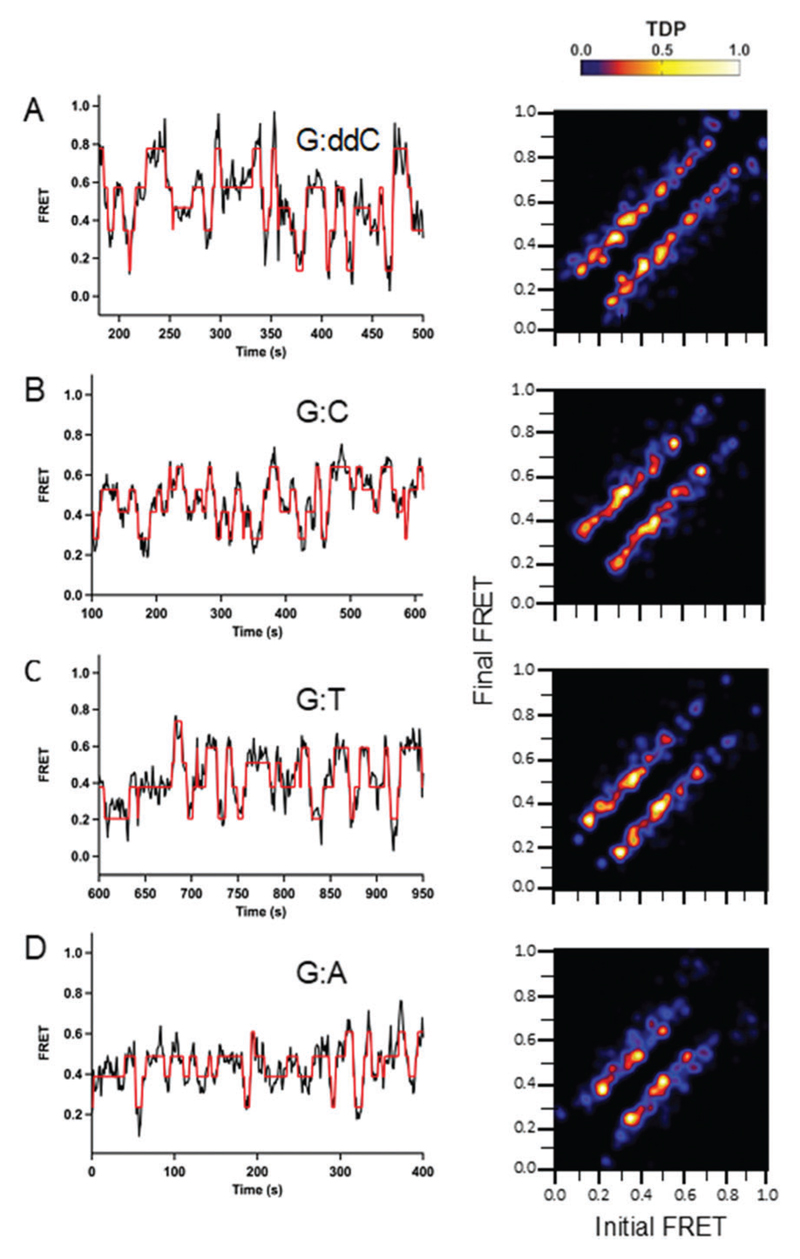
Conformational dynamics observed for wild type Pol III core as a function of varying DNA termini. Single-molecule experiments were performed in 20 mM Hepes, pH 7.6, 5 mM potassium glutamate, 3 mM magnesium acetate, 2 mM DTT, 2 mM Trolox, 25 nM enzyme and 10 μM dCTP. For the G:ddC DNA, a dideoxyC was inserted at the 3′ primer end to prevent nucleotide insertion. For the G:C DNA, a non-hydrolyzable dCTP analog containing an (α,β)-methylene bridge was utilized to prevent nucleotide incorporation. For the mismatches G:T and G:A, a phosphorothioate modification was incorporated into the primer strand to prevent degradation (see Table 1 for DNA sequences). Long Pol III core binding (>60 s) smFRET trajectories were analyzed using a hidden Markov model with the freely available HaMMy software. FRET trajectories were compiled into transition density plots (TDP) that depict the number of times a transition occurs as a two-dimensional heat map containing the initial and final FRET values on the *x* and *y* axis, respectively. (A, *n* = 33; B, *n* = 37; C, *n* = 42; D, *n* = 51.)

**Fig. 3:**
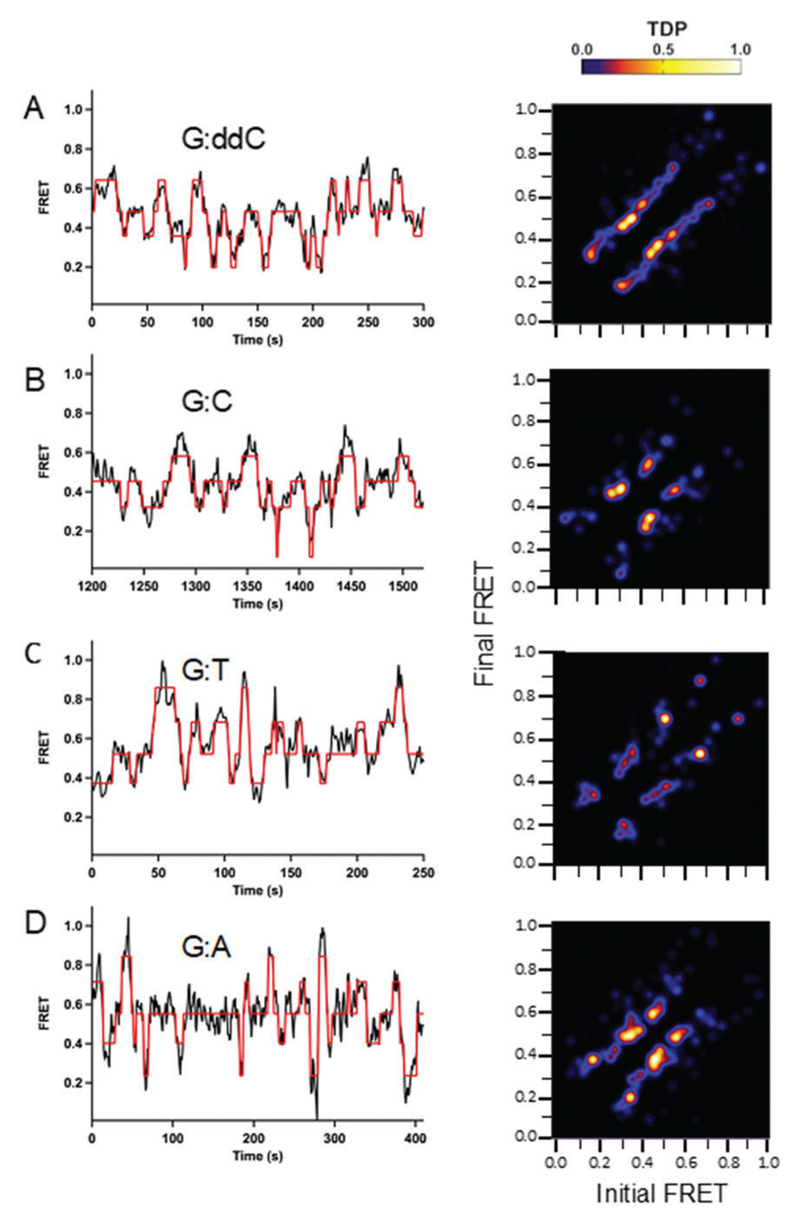
Conformational dynamics observed for mutant Pol III core containing a Y453A mutation in the thumb domain of the α-subunit as a function of varying DNA termini. Single-molecule experiments were performed in 20 mM Hepes, pH 7.6, 5 mM potassium glutamate, 3 mM magnesium acetate, 2 mM DTT, 2 mM Trolox, 25 nM enzyme and 10 μM dCTP. For the G:ddC DNA, a dideoxyC was inserted at the 3′ primer end to prevent nucleotide insertion. For the G:C DNA, a non-hydrolyzable dCTP analog containing an (α,β)-methylene bridge was utilized to prevent nucleotide incorporation. For the mismatches G:T and G:A, a phosphorothioate modification was incorporated into the primer strand to prevent degradation (see Table 1 for DNA sequences). Long Pol III core binding (>60 s) smFRET trajectories were analyzed using a hidden Markov model with the freely available HaMMy software. FRET trajectories were compiled into transition density plots (TDP) that depict the number of times a transition occurs as a two-dimensional heat map containing the initial and final FRET values on the *x* and *y* axis, respectively. (A, *n* = 19; B, *n* = 10; C, *n* = 12; D, *n* = 20).

A set of smFRET trajectories showed dynamic transitions within a long (>60 s) single binding event. These trajectories reveal information about the conformational pathway that Pol III core adopts along the Pol- and Exo-binding landscape. A Hidden Markov Model analysis (HMM) was performed on these smFRET trajectories using the program HaMMy.^22^ From the HMM fits, transition density plots (TDPs) were generated using the program TDP.^22^ TDPs are two-dimensional graphs in which the initial and final FRET states are plotted as a heat map. While shorter Pol III core binding events also displayed dynamic transitions, these data were not included in the analysis, as more data points are needed for HMM. The initial FRET value is shown at the *x*-axis, and the final FRET value, after the transition, is depicted on the *y*-axis. Here, higher probability FRET states are represented by a yellow-white color and lower probability states by a blue-red color (see legend heat map, Fig. 2 and 3 and Methods for how TDP plots were generated).

The transitions for all TDP plots are symmetric in both directions, demonstrating reversibility in switching between the different FRET states. Overall, the TDP transitions show that wild type Pol III core binds over the entire FRET range between 0.2 and 0.8 and that its binding is highly dynamic. The highest number of transitions observed for all DNA substrates was for G:ddC (Fig. 2A). Further, it has the highest density of transitions between 0.4–0.6 FRET efficiencies. In addition, compared to the other DNA pairs tested, G:ddC populates the 0.8 FRET state with the highest density (Fig. 2A). For G:C, the highest density of transitions is between 0.4–0.6 (Fig. 2B). In case of the mismatches G:T and G:A, the transition densities are highest between 0.2–0.6 (Fig. 2C and D, respectively). Finally, a reduction in densities for the higher FRET states, 0.6–0.8, are reduced for the mismatches in comparison to the cognate pairs G:ddC and G:C; this is especially apparent for the G:A mismatch. While we can observe differences in wild type proofreading dynamics by changing the molecular identity of the terminal base pair, dynamics in proofreading at the protein level are limited using this approach. Therefore, we examined dynamics with an Exo-deficient Pol III mutant.

### Mutant Pol III has similar binding kinetics as wild type but decreased conformational dynamics

We hypothesize that the large degree of conformational dynamics for wild type Pol III core arise from proofreading. To test this, smFRET studies with the Pol III core mutant containing a Y453A mutation in the polymerase (α) subunit were performed. This mutation removes a tyrosine residue within the thumb domain of Pol III that is suggested to aromatically stack with a nucleotide near the 3′ end on the primer strand, thereby stabilizing Exo-binding mode.^14^ Moreover, this mutant has previously been reported to have strongly reduced exonuclease activity.^14^

The binding rate constants were determined for the mutant Pol III and, overall, no significant differences were determined in comparison to wild type (Fig. S5, ESI†). For example, the off rate constants for the mutant ranged from 0.3–0.4 s^−1^ (Table 2); which is similar to those determined for wild type Pol III. Further, the *k*_on,slow_ for the mutant did not show appreciable differences amongst the matched and mismatched DNA terminal base pairs. One difference, however, was for the *k*_on,fast_ for the mutant and G:A (1.5 s^−1^, Table 1), which was 5 times faster than for the wild type (0.3 s^−1^, Table 1).

Significant differences in conformational dynamics for the wild type and mutant were determined from the HMM analysis (Fig. 2 and 3, respectively). For the mutant Pol III core, the number of transitions from 0.2–0.8 for the G:ddC DNA is markedly reduced in comparison to the wild type protein (Fig. 3A and 2A, respectively). However, the highest transition densities are still between 0.4–06 for both the mutant and wild type Pol III core for the G:ddC DNA. In the case of G:C, two dominant transition densities are present around 0.4 and 0.6 FRET efficiencies (Fig. 3B). Additionally, a reduction in the transition densities is observed for the lower (0.2) and higher (0.7–0.8) FRET states in the case of the mutant Pol III core as compared with the wild type for the G:C DNA (Fig. 3B and 2B, respectively). For the G:T mismatch, the highest density transition is present at 0.7 and for G:A it occurs at 0.2, 0.4 and 0.6 FRET states (Fig. 3C and D, respectively).

### Pol III core mutant has reduced conformational flexibility

**Fig. 4:**
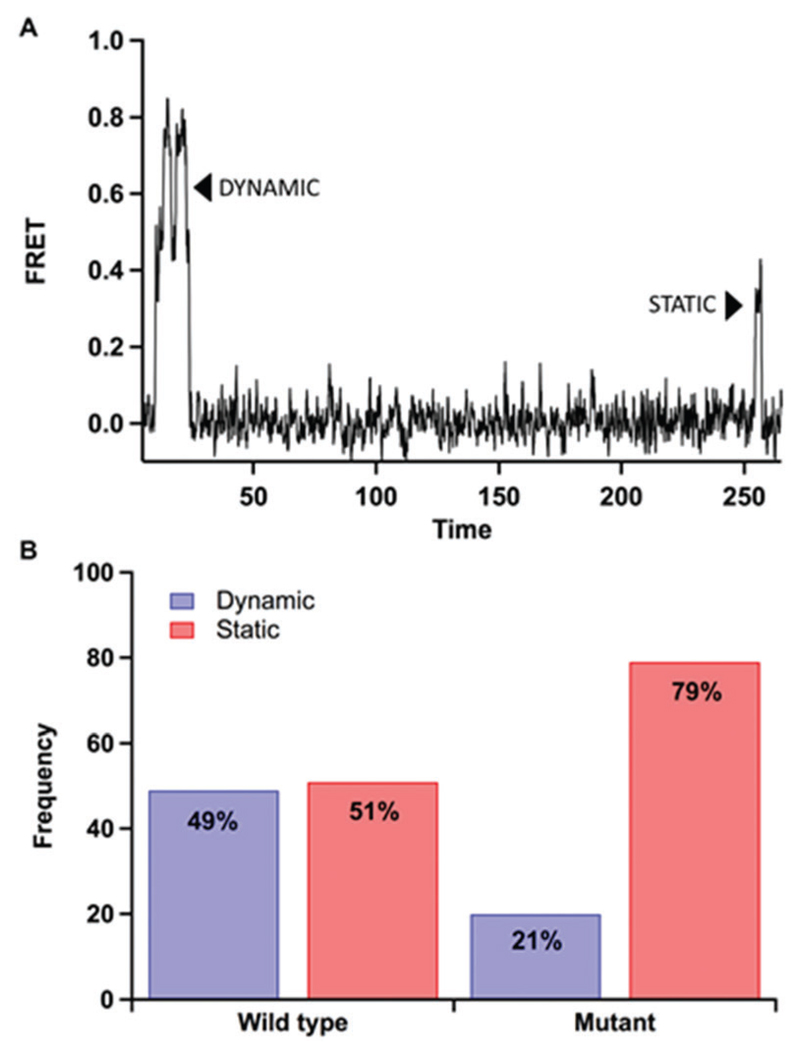
Comparison of dynamic *versus* static binding events for wild type and Pol III core mutant containing a Y453A mutation in the thumb domain of the α-subunit using the method of maximum likelihood analysis for the G:AA double mismatch. Single-molecule experiments were performed in 20 mM Hepes, pH 7.6, 5 mM potassium glutamate, 3 mM magnesium acetate, 2 mM DTT, 2 mM Trolox, 25 nM enzyme and 10 μM dCTP. (A) Example of a dynamic event (left) that has distinct changes in FRET over the course of the binding event and a static event (right) that has one FRET state through the duration of the binding event. (B) Frequency of dynamic and static binding events for wild type (*n* = 100) and mutant (*n* = 96) DNA Pol III. Here, the wild type has a higher frequency of dynamic events (49%) than the mutant (21%) as shown in blue.

The frequency of static and dynamic binding events were quantified using the method of maximum likelihood analysis at a double DNA mismatch (Table 1, G:AA sequence). Here, traces were classified as either static, in instances where no change in FRET was observed during a bind event, or dynamic, in instances were multiple FRET states were observed during a single binding event (Fig. 4A). Dynamic events were considered to be a change in FRET of 0.1 or more for at least a 50 ms time duration. For the wild type protein, a similar distribution in dynamic and static events was quantified, 49% and 51%, respectively (Fig. 4B). For the mutant, a significant reduction in the dynamic events was observed in comparison to the static events, 21% and 79%, respectively (Fig. 4B).

**Fig. 5:**
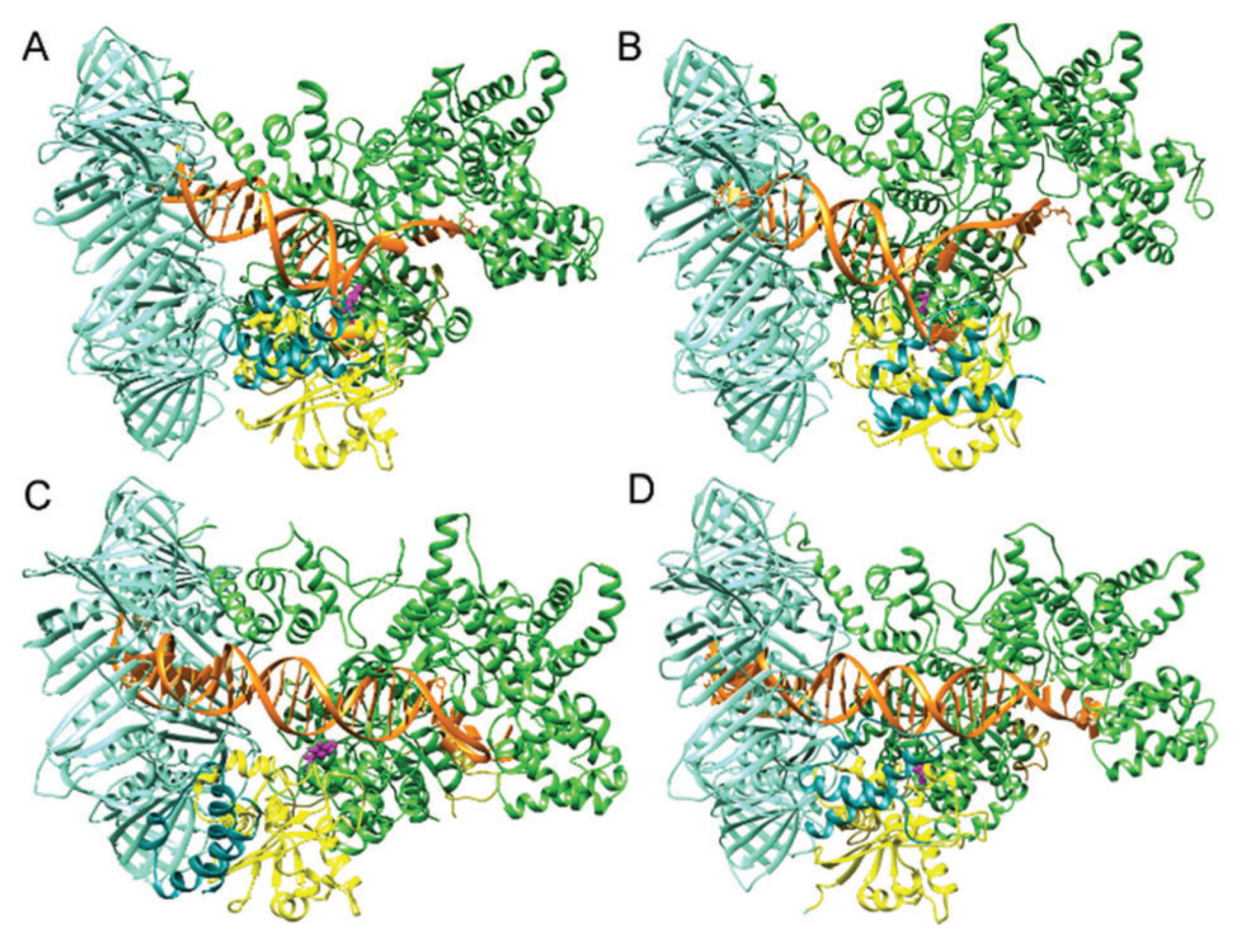
Representative snapshots from the molecular dynamics simulations for (A) Exo mode, (B) Exo mode mutant, (C) Pol-dC, and (D) Pol-dC mutant. Mutation site is highlighted in pink.

Molecular dynamics (MD) simulations were performed to gain atomic-level insights into the dynamics of the wild-type and mutant systems. These simulations also showed more Pol III motions for the wild type as compared to the mutant (Fig. 5). Eight representative systems were constructed for a Pol III core-clamp model (α, β, ε, θ complex, see Methods section) for wild type systems in the Exo mode, Pol mode with correct dG-dC matched base at the DNA terminus (Fig. S9, ESI†), Pol mode with dG–dA mismatched base at the DNA terminus, and an apo structure with no DNA. A representative image indicating each subdomain is seen in Fig. S9 (ESI†). Each of these was also mutated at 453 from Tyr to Ala for another set of systems. These simulations ran for a comparatively short time, 225 nanoseconds (ns) for each system (1.8 μs total), compared with the FRET experiments due to the constraints of computer hardware and the large size of the system (~400 000 atoms including solvent). Overall, interesting correlations between the simulation data and single-molecule experimental results were obtained.

Distances between the site of the Cy5 acceptor (tag location, residue 41 on the θ subunit) and the site on the DNA (alpha C) for the Cy3 donor were calculated over time for each trajectory (Fig. S9, ESI†). The Exo modes show a distance of around 35–40 Å, while the Pol modes show a distance from 50–65 Å. The Pol-dC wild type shows the highest amount of distance and conformational variation over time for the tag distance, spanning a range of ~50–70 Å. Comparatively, the Pol-dC mutant structure shows a reduction in overall distance with a large drop at 70 000 frames (140 ns) (Fig. S10, ESI†). This decrease is caused by the DNA rapidly sliding down, which could in turn have resulted from changes in the hydrogen bonding from the mutation. However, it was observed as an isolated event, and could be an artifact of that single simulation, since this was not observed in the other mutant trajectories. The tag distance variation for wild type Pol-dA is consistent between 52–60 Å and has a substantial decrease in fluctuation compared with Pol-dC, consistent with the smFRET experimental results. The wild type Exo and Exo mutant modes span about 30–40 Å and vary less compared to the Pol-dC and Pol-dC mutant modes, again consistent with the single-molecule FRET data.

**Fig. 6:**
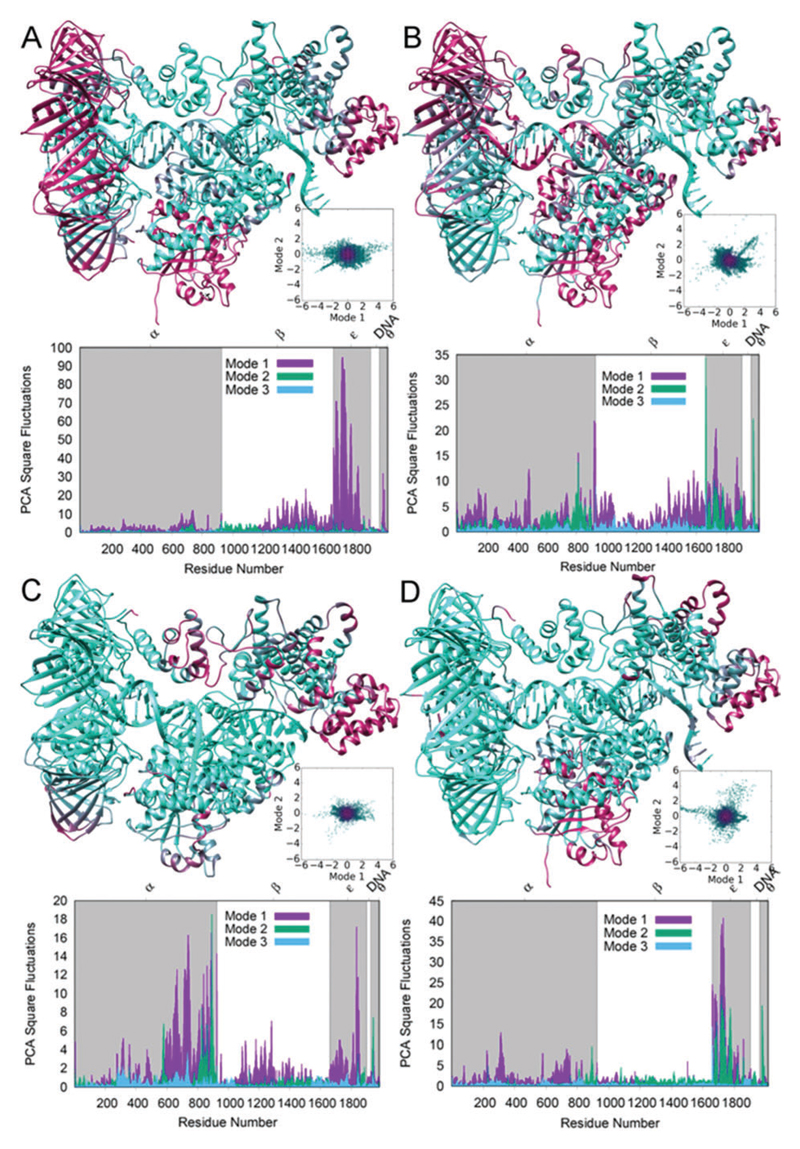
Square fluctuations for principal normal modes displayed in order to show differences in top protein vibrational modes and differences in dynamic movements. 3D structures colored by relative amount of movement from most (mauve) to least (cyan) of normal mode 1. Scatterplots are of normal mode 1 (PCA1) *versus* normal mode 2 (PCA2). Graph shows per-residue square fluctuations for principal modes 1, 2 and 3 labeled by residue range. (A) Polymerase mode with correct terminal primer base pair (G:C) (Pol-dC). (B) Polymerase mode with the incorrect terminal primer base pair (G:A) (Pol-dA). (C) Editing mode with the primer positioned in the proofreading domain (Exo-dA). (D) Polymerase mode with the correct terminal primer base pair (G:C) and the Y453A mutant (Pol-dC mutant).

To investigate the difference in motion and conformation between wild type and mutant Pol III and with the matched and mismatched termini, we performed principal component analysis (Fig. 6). Principal component analysis is a convenient way to assess relative differences in dynamics and overall motion between systems, and to hone in on specific differences in protein vibrational motion. In this case, the differences in overall structure indicate stable systems and relatively small structural shifts between the systems (Fig. S11–S16, ESI†), but substantial differences in dynamic motion. The first principal component analysis normal mode (PCA1) is shown mapped onto a three-dimensional structure. Further, per residue square fluctuations for PCA1–PCA3 are depicted on the major graph for each panel, and show that the majority of the vibrational motion is contained in the first two modes. There are areas of high fluctuation primarily in the β_2_-clamp, ε and part of the finger domain in α for Pol-dC (Fig. 6A). For Pol-dA (Fig. 6B), a decrease in square fluctuations appears in the β_2_-clamp domain, a shifting of location for fluctuations in the finger domain, an increase in ε (Exo domain) and an increase for the primer strand of DNA with the mispaired base. While the locations of the square fluctuations spread out more for Pol-dA as compared to Pol-dC, the amount of fluctuation decreases fairly substantially (Fig. 6B). The Exo mode shows an almost complete loss of fluctuation in the clamp, a decrease in the theta domain and an increase in the alpha and epsilon domains as compared to the Pol modes (Fig. 6C).

The Pol-dC mutant shows a substantial decrease in overall square fluctuations in the first two normal modes, although the locations of high fluctuation remain similar (Fig. 6D). Similarly, the Pol-dA mutant (Fig. S17E, ESI†) shows similar locations of fluctuation, but an overall decrease in square fluctuations for the first two modes. The Apo structure without DNA (Fig. S18G, ESI†) and Apo mutant (Fig. S18H, ESI†) also show large changes in the first few normal modes, which correlates with the smFRET experimental data showing that the Y453A mutation reduces the dynamics of Pol III core.

## Discussion

DNA Polymerases are responsible for accurately replicating genetic information. To increase replication fidelity, many DNA Pols have proofreading activity that removes incorrectly inserted nucleotides. This study examined proofreading dynamics for *E. coli* Pol III core in the presence of the β_2_-sliding clamp at varying DNA mismatches. To investigate proofreading dynamics, we developed a smFRET assay that monitors the binding rate constants and conformational changes that Pol III core adopts on DNA containing matched and mismatched terminal base pairs as well as performed MD simulations on a Pol III core model to understand the dynamics for Pol III core. For the smFRET assay, a Cy5 acceptor fluorophore was synthetically incorporated on to a genetically modified cysteine analog of the θ subunit of Pol III. This ensured a single acceptor label on the protein and a single donor label on our DNA, in order to accurately determine and assign FRET values in our assay.

Previous studies involving *E. coli* DNA polymerases have revealed new insights into polymerization and proofreading dynamics.^23^–^26^ For example, a recent single-molecule flow-based study with Pol III core showed that transfer of the primer strand during proofreading did not disrupt interactions between the ε and β_2_ subunits.^27^ Further, this work showed that the ε and β_2_ interaction remains intact in Pol and Exo mode. Also, a co-localization single-molecule spectroscopy assay that monitored loading of *E. coli* proteins during replication did not observe changes in lifetimes of the Pol III core in presence of mismatched DNA or an *N*^2^-furfuryl-dG lesion.^28^ Similarly, we did not observe a change in binding kinetic rate constants of the Pol III core in the presence of matched or mismatched DNA termini.

A significant contrast in the conformational dynamics during proofreading is observed for DNA Pol I and DNA Pol III. Work with *E. coli* DNA Pol I determined the kinetic rate constants for active site switching between the Pol and Exo domains; there, a smFRET assay was developed containing a Cy5-labeled DNA Pol I and Cy3-labeled DNA.^29^ For Pol I, two FRET states were assigned, one for the Pol-binding mode at 0.82 and another at 0.67 for the Exo-binding orientation. Interestingly, there is a significant difference in the dynamics for DNA Pol I compared to Pol III core. For Pol III core, our transition density plots analysis shows that Pol III binds very dynamically to DNA and more than two FRET states are observed (Fig. 2 and 3), unlike for DNA Pol I. This highlights a contrasting dynamic range for these two polymerases. It is important to note that DNA Pol I and Pol III belong to different polymerase families (A and C-type, respectively) and have their exonuclease domain located in very different positions, which could contribute to the differences in proofreading dynamics. In addition, the smFRET assay with Pol III core is in the presence of the sliding clamp, which could also have an influence on the binding dynamics and lead to an increase in the number of intermediates along the proofreading pathway in comparison to the single protein DNA Pol I.

It was surprising to observe that the matched base pairs (G:C and G:ddC) revealed a higher number of conformational dynamics than for the mismatches (G:T and G:A), shown in the TDP analysis (Fig. 2 and 3) and the MD simulations (Fig. 6). These dynamic differences were also observed in MD simulations where the measured tag distances for the Pol-dA system had a substantial decrease in fluctuation compared with the Pol-dC system (Fig. S10, ESI†). The observed experimental difference in dynamics may be explained by the fact that system is chemically modified to prevent polymerase-mediated elongation (*i.e.* dideoxy and non-hydrolyzable dCTP). Moreover, these chemical modifications could lead to an enzyme that is probing a variety of conformations to mitigate the disturbance and effectively try to push the forward replication reaction to proceed. In the case of the mismatch, however, it is possible that the protein takes longer while partitioning to the exonuclease domain for proofreading to occur. Indeed, since the MD simulations do not contain the fluorescent organic dyes during the calculations, an alternative explanation for this observation is that these dynamics are attributed to the natural behavior of the protein.

In addition to a difference in dynamics as a function of the terminal base pair, we also observed a varied range of dynamics for the wild type compared to the mutant. This effect is observed by comparing the wild type and mutant TDP plots for each respective DNA (Fig. 2 *vs.* Fig. 3). In addition, we compared the number of dynamic and static events for wild type and mutant Pol III core binding to DNA with a terminal double mismatch (Fig. 4). Here, we found that wild type Pol III has a relatively equal distribution of dynamic and static events at a (49% and 51%, respectively). While, for the mutant, we observed a significant reduction in the number of dynamic events (Fig. 4). In addition, the simulation data monitoring tag distances show that the Pol-dC mutant has a reduction in overall distance with a large drop at 70 000 frames (140 ns) (Fig. S10, ESI†). This could be due to a DNA slipping mechanism arising from the mutation from tyrosine to alanine reducing the stability of the DNA in the active site (Fig. S10, ESI†). This difference in Pol *vs.* Exo mode dynamics has also been reported for DNA PolB in *Pyrococcus furiosus*.^30^ In MD simulations, PolB showed a change in conformational dynamics in the Exo mode compared with the Pol mode and it is hypothesized that interactions between PolB and the clamp subunit contributes to the observed change in dynamics compared to the Exo mode.

To further investigate the influence of the mutation, correlation analysis was performed between the Exo wild type and Exo mutant structure (Fig. S19, ESI†). The locations of increased correlation and anticorrelation are similar to that shown in the principal component analysis of the normal modes, with the majority of anticorrelation difference on the DNA and finger domain and the majority of the correlation difference on θ, ε and the finger domain of α. Though the mutation results in a clear change in the overall DNA location and structure, the DNA base remains stable in the exonuclease active site, with the majority of anticorrelation occurring near the mutation location, as one might expect. Further, overall distance changes between Y453 and A453 of ~4–5.6 Å were observed, suggesting the mutation alters the local positioning of the α helix of the protein (Fig. S19–S21, ESI†). These results are consistent with the experimental finding of relatively similar activity despite the mutation, but with a change in the overall dynamics. Correlation analyses of the Pol mode show similar results, with the strongest differences in correlation between wild type and variant appearing with a mismatched base pair in Pol mode (Fig. S20 and S21, ESI†).

**Fig. 7:**
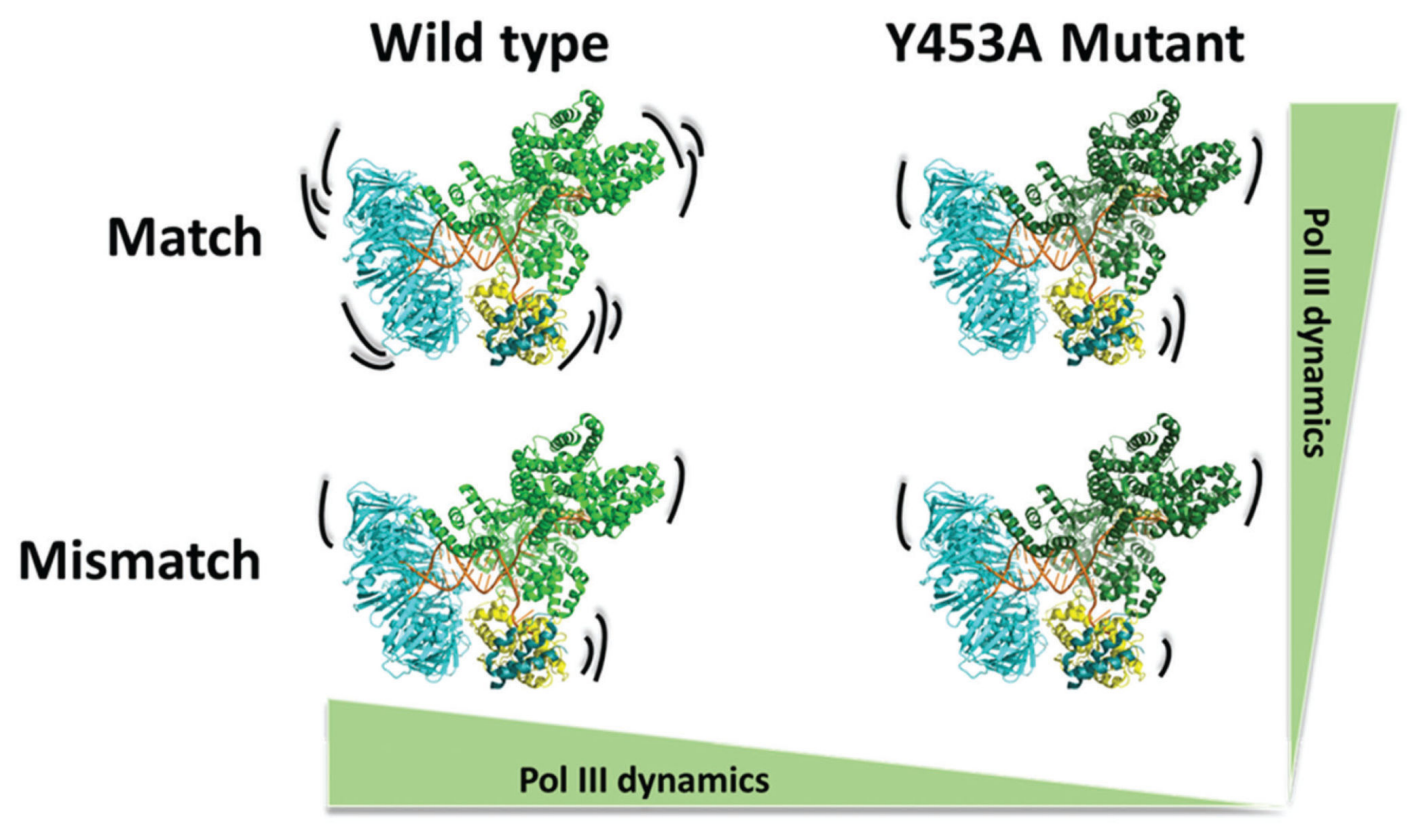
The conformational dynamic range of Pol III core varies as a function of the terminal DNA structure, *i.e.* a matched or mismatched base pair, as well as for the wild type Pol III core and the exonuclease deficient mutant with a Y453A mutation in the thumb domain. This demonstrates two key molecular features in which the Pol III core dynamics can be influenced (1) the terminal structure of the DNA base pairs and (2) the tyrosine residue 453 within the thumb domain of the α subunit.

This study reveals a model for the relative conformational dynamics of Pol III core. The present data indicate two main findings. First, wild type Pol III core is more dynamic than the exonuclease deficient Y453A mutant. Second, that matched base pairs evoke higher Pol III dynamics than with mismatched DNA termini (Fig. 7). Regarding the observed reduction in conformational flexibility for the Y453A mutant, this suggests that this tyrosine residue influences the structural and conformational dynamics that are involved in Pol III core proof-reading. Structural and simulation data suggests that Y453 stabilizes Exo mode by aromatically stacking with a nucleobase on the primer strand. Our data shows that the loss of this tyrosine lowers the overall protein dynamics of Pol III core and we hypothesize that the loss of this tyrosine moiety removes a stabilization factor necessary for efficient proofreading. Further studies that monitor DNA polymerase dynamics with mutated residues that stabilize and destabilize Exo mode are needed to determine their influence on proofreading and, ultimately, their role in mutagenesis and carcinogenesis in humans.^6^

## Materials and methods

### DNA labeling and purification

DNA constructs were purchased from IDT Technologies containing 5′ biotin TEG for the template DNA and an amino-modified dT-C6 for labeling with a Cy3 NHS ester from GE Healthcare. For the mismatches G:T and G:A, a phosphorothioate modification was incorporated into the primer strand to prevent degradation during the single-molecule imaging experiments. Phosphorothioate modifications were not used with the matched DNA, as we do not expect exonuclease activity on matched DNA ends. Labeling reactions were carried out according to manufacturer protocols. Briefly, labeling reactions were performed with 16 nmoles of DNA in 0.1 M Na_2_CO_3_, pH 9.0. To this, 7 μl the mono-reactive Cy3 dye vial was added, which was previously suspended in 14 μl of DMSO. The reaction was performed overnight at 27 °C. Next, DNA was ethanol precipitated, resuspended in DNase free water and purified on a reverse phase C8-column (Sigma-Aldrich Supelco Discovery BIO wide pore C8, 25 cm × 4.6 mm × 5 μm) with a gradient of 50 mM TEAA and acetonitrile. The desired labeled DNA fractions were collected, desalted by ethanol precipitation and stores at −20 °C until needed.

### Protein expression, purification and labeling

All proteins were expressed in *E. coli* BL21 (DE3).^14^,^18^ Enhanced clamp binding mutants of polymerase and exonuclease were used with amino acid changes for the polymerase in residues QADMF to QLDLF from 920–924 and for the exonuclease QTSMAF to QLSLPL from 182–187.^14^ Pol IIIα, β and ε subunits were expressed and purified as previously described^19^ and were flash frozen in liquid nitrogen and stored at −80 °C. θ mutant E41C was created to incorporate a Cys residue for maleimide labeling. The Quikchange lightning kit (Agilent) was used for site-directed mutagenesis. θ was purified on a Histrap HP column and a Superdex 75 gel filtration column. The θ subunit containing a E41C mutation was labeled with Cy5 maleimide (GE Healthcare) following manufacturer instructions. Pol III core complexes consisting of α, β and ε were assembled at equistoi-chiometric ratio and then chromatographically separated by gel filtration (Superdex 75 column). Proteins were flash frozen in liquid nitrogen and stored at −80 °C until needed.

### Single-molecule measurements

Single-molecule experiments were performed as previously described.^15^,^16^ Briefly, quartz slides and cover slips were prepared as previously described. Briefly, slides were passivated with methoxy-PEG-SVA (*M*_r_ 5000, Laysan Bio Inc.) along with 10% biotin-PEG-SVA (*M*_r_ 3400, Laysan Bio Inc.) in 0.1 M NaHCO_3_. For imaging, slides were first incubated with 0.2 mg ml^−1^ BSA (Sigma-Aldrich) in T50 buffer (10 mM Tris-HCl, pH 7.0 and 50 mM NaCl) for 10 min. BSA was then washed with T50 buffer and neutravidin (0.2 mg ml^−1^ in T50 buffer) was applied and incubated for 10 min. Excess neutravidin was removed with Pol buffer A (20 mM Hepes, pH 7.6, 5 mM potassium glutamate, 3 mM magnesium acetate, 2 mM DTT and 2 mM Trolox). Then, DNA was surface-immobilized by injecting a 25 pM solution of pre-annealed primer–template DNA in Pol buffer A and incubating for 10 min. Excess DNA was removed with imaging buffer containing the protein (25 nM) in Pol buffer A, and either 10 μM dCTP or, in the case of the G:C DNA, a non-hydrolyzable dCTP analog (Cytidine-5′-[(α,β)-methyleno]triphosphate) (Jena Bioscience) and an oxygen scavenging system containing 5 mM 3,4 dihydroxybenzoic acid (PCA) and 60 nM protocatechuate dioxygenase (PCD) (Sigma-Aldrich). DNA was then imaged on a home-built prism-based total internal reflection microscope. Imaging was performed at room temperature. A continuous green excitation (532 nm) laser was applied at 1.5 mW power and 80 ms time resolution for smFRET experiments. Apparent FRET efficiencies were calculated as FRET = *I*_A_/(*I*_D_ + *I*_A_); and *I*_A_ indicates acceptor intensity and *I*_D_ indicates donor intensity. The donor emission leaking into the acceptor channel was corrected for by applying a 10% correction factor. For data analysis a custom written software script for IDL and Matlab was used. Single-molecule trajectories were analyzed with seven-point averaging and then binned to generate a FRET histogram for each analyzed trace. Then, a composite FRET histogram was compiled from multiple trajectories with IGOR Pro version 6 (WaveMetrics).

### Hidden Markov Model and TDP analysis

Long Pol III core binding (>60 s) smFRET trajectories were analyzed using a hidden Markov model, described previously.^22^ Only FRET efficiencies between 0.05 and 1.0 within the entire length of the trace were analyzed. Data was fit using a five-state model with the freely available HaMMy software. FRET trajectories were compiled into transition density plots that depict the number of times a transition occurs as a two-dimensional heat map containing the initial and final FRET values on the *x* and *y* axis, respectively.

### Dwell time analysis

Kinetic rate constants *k*_on_ and *k*_off_ (s^−1^) were determined by a threshold-based dwell time analysis using custom written software in Matlab, as described.^15^,^16^ The on dwell times were calculated from the times between binding events (*t*_on_), used to determine the *k*_off_, while the off dwell times were calculated as the time that Pol III core was bound to the DNA (*t*_off_), and used to determine *k*_on_. Kinetic curves were generated in Igor Pro software and fit to single (eqn (1)) or double (eqn (2)) exponential decay equations. Here, *f* (*t*) is the fraction of protein bound after time (*t*), *A* (*i.e. A*, *A*_1_ or *A*_2_) are the amplitudes of the phases and *k* (*i.e. k*, *k*_1_ or *k*_2_) are the rate constants for protein binding to the DNA.

(1) f(t)=Aexp(−kt)

(2) $$f\left(t\right)=A_{1}\;\text{exp}\left(-k_{1}t\right)+A_{2}\exp\left(-k_{2}t\right)$$

### Primer extension and degradation assay

Experiments were performed in 10 μl reactions with 10 nM of pre-annealed primer–template DNA. DNA primers were 5′-end labeled with Cy3. Reaction mixtures were incubated at 25 °C containing 25 nM protein, 20 mM Hepes (pH 7.6), 5 mM potassium glutamate, 3 mM magnesium acetate, 2 mM DTT and 30 μg ml^−1^ BSA. Reactions were terminated by adding 10 μl of 95% formamide, 35 mM EDTA and 0.1% bromophenol blue at varying times (stated in the figures). Gels were visualized on a Typhoon FLA 7000 instrument (GE Healthcare).

### Method of maximum likelihood analysis

To quantify single-molecule trajectories as static or dynamic the method of maximum likelihood analysis was performed. Here, individual smFRET trajectories were loaded into a home-written Matlab script (courtesy of Julie Biteen).^31^ Parameters for dynamic traces were applied to include any change in FRET ≥0.1 for a duration of at least 50 ms. Static events were considered binding events with no change in FRET using the criteria indicated for the dynamic classification. Here, each binding event was classified as either static or dynamic and quantified for both the wild type and mutant polymerase.

### Computational MD simulations

Cryo-EM structures for the replicative (PDB 5FKW)^18^ and Exo (PDB 5M1S)18 modes were used as templates and individual subunits were compared against reported crystal structures to ensure the residue locations were correct to within ~1 Å for the β_2_-clamp (PDB 1MMI)^32^ α (PDB 2HNH)^33^ and ε (PDB 2GUI, to be published) domains by using Chimera’s structure comparison. Chimera is developed by the Resource for Biocomputing, Visualization, and Informatics at the University of California, San Francisco (supported by NIGMS P41-GM103311).^34^ Eight total systems were created, four in the Pol mode with correct/incorrect base pairings at the primer/template terminus and with/without the Y453A mutation. Two more systems were created in the proofreading mode with and without the mutation, and two Apo structures were created with and without the Y453 mutation. The Mn^2+^ metal ions were placed based on the location from the 2GUI structure into analogous locations for the ε domain in the proofreading mode, based partially on our previous work.^35^ For the Pol mode, Mg^2+^ metal ions were placed in the α subunit from analyzing homologous crystal structures, and the θ domain was added from the Exo mode. Short 5 ns simulations were performed to ensure stability of metal locations for both Exo and Pol modes. We also performed two 5 ns simulations without the metal ions, which results in unstable DNA in the active site. Missing residues were replaced using Modeller,^36^ and the structures were protonated and checked using Molprobity.^37^ The DNA chains were rebuilt and slightly extended in accordance with the experimental DNA sequence, with systems created in the Pol mode with the terminal primer base at the primer/template terminus as matching (dC) and mismatching (dA) bases. Each system was solvated with a minimum distance of 12 Å from the edge of the protein to the edge of the box, and neutralized with 117 K^+^ counterions added to Pol systems, 99 added to Exo systems, and 63 added to apo systems. All simulations were performed using the AMBER ff14SB force field for the protein and DNA parameters,^38^,^39^ with Mn^2+^ parameters taken from the literature.^40^ Production was run in NVE with the AMBER’s pmemd.cuda program for 225 nanoseconds for each system after initial density equilibration at constant pressure and slow heating to 300 K at constant volume.^41^ A 2 fs timestep was used with sPME for long-range electrostatics and an 8 Å non-bonded cutoff.^42^ Hydrogen atoms were constrained using SHAKE.^43^ Amber’s Cpptraj program was used to perform the majority of the statistical analysis, aside from the normal modes and principal component analysis, which was performed using VMD’s ProDy Normal Mode Wizard plugin.^44^,^45^

## Supplementary Material

† Electronic supplementary information (ESI) available: Additional exponential decay curves, primer extension data, distribution plots and MD simulated results. See DOI: 10.1039/c8cp04112a

Supp Materials

## Figures and Tables

**Table 1:** DNA sequences


DNA	Sequence^a^

G:ddC	5′ CATAATATCC TCAGGAGTCC TTCGTCCTAG TACTACTCA 3′ *c*TCAGG AAGCAGGATC ATGATGAGT 5′
G:C	5′ CATAATATCC TCAGGAGTCC TTCGTCCTAG TACTACTCA 3′ CTCAGG AAGCAGGATC ATGATGAGT 5′
G:T	5′ CATAATATCC TCAGGAGTCC TTCGTCCTAG TACTACTCA 3′ TTCAGG AAGCAGGATC ATGATGAGT 5′
G:A	5′ CATAATATCC TCAGGAGTCC TTCGTCCTAG TACTACTCA 3′ ATCAGG AAGCAGGATC ATGATGAGT 5′
G:AA	5′ CATAATATCC TCAGGAGTCC TTCGTCCTAG TACTACTCA 3′ AACAGG AAGCAGGATC ATGATGAGT 5′

a Template (top) and primer (bottom) strands. Bold T denotes Cy3-labeled amino-dT linker, lower case *c* denotes dideoxy cytosine terminated primer, underlined bases contain a phosphorothioate bond to prevent exonuclease cleavage. All 39-mer template strands contain a 5′ -Biotin TEG for surface immobilization.

**Table 2:** Binding rate constants for wild type and mutant Pol III core


DNA^a^	*k*_on,fast_ (s^−1^)	*k*_on,slow_ (s^−1^)	*k*_off_ (s^−1^)	*N*

Wild type				
G:ddC	0.8 ± 0.1	0.030 ± 0.010	0.20 ± 0.01	104
G:C	0.6 ± 0.1	0.020 ± 0.007	0.20 ± 0.01	94
G:T	0.6 ± 0.1	0.010 ± 0.003	0.20 ± 0.01	103
G:A	0.3 ± 0.1	0.020 ± 0.002	0.20 ± 0.01	102
G:AA	N.D.	N.D.	0.30 ± 0.02	107
Mutant^b^				
G:ddC	0.7 ± 0.1	0.05 ± 0.01	0.40 ± 0.02	99
G:C	0.8 ± 0.1	0.04 ± 0.01	0.30 ± 0.01	141
G:T	0.6 ± 0.1	0.05 ± 0.01	0.30 ± 0.01	104
G:A	1.5 ± 0.3	0.04 ± 0.01	0.30 ± 0.02	143
G:AA	N.D.	N.D.	0.30 ± 0.02	104

a DNA sequences in Table 1.

b Mutant is a Y453A modification in the α subunit.
