# Supplementary material for: Reduced structural flexibility for an exonuclease deficient DNA polymerase III mutant†
Source: Phys Chem Chem Phys. Author manuscript; Available in PMC 2018 Dec 4. (PMC6278910; doi:10.1039/c8cp04112a)
Supplement: Supp Materials [file NIHMS80623-supplement-Supp_Materials.pdf]

This journal is © the Owner Societies 2018

Reduced structural flexibility for exonuclease deficient DNA polymerase III mutant

Hailey L. Gahlon[1,2], Alice R. Walker[3], G. Andrés Cisneros[3], Meindert H. Lamers[4] and David S. Rueda[1,2]*

[1] Department of Medicine, Molecular Virology, Imperial College London, Du Cane Road, London W12 0NN, UK. [2] Single Molecule Imaging Group, MRC London Institute for Medical Sciences, Imperial College London, Du Cane Road, London, W12 0NN, UK. [3] Department of Chemistry, University of North Texas, 1155 Union Circle, Denton, Texas 76203, United States. [4] MRC laboratory of Molecular Biology, Francis Crick Avenue, Cambridge Biomedical Campus, Cambridge, CB2 0QH, United Kingdom.

* To whom correspondence should be addressed. Tel: +44 (0)20 3313 1604; Email: david.rueda@imperial.ac.uk

Present Address: Meindert H. Lamers, Leiden University Medical Center, Leiden, 2333 ZC, The Netherlands

Figure S1. Polyacrylamide gel analysis of θ E41C protein S2

Figure S2. Time course primer extension and degradation assay S2

Figure S3. Binding exponential decay curves for wild type Pol III S3

Figure S4. Binding exponential decay curves at a double mismatch S3

Figure S5. Binding exponential decay curves for mutant Pol III S4

Figure S6. Histograms of canonical (G:ddC and G:C) termini S5

Figure S7. Histograms of mismatched (G:T and G:A) termini S5

Figure S8. Histograms of double mismatched (G:AA) DNA S6

Figure S9. Schematic of overall structure of Pol III in the building mode S6

Figure S10. Tag distance simulations S7

Figures S11-16. RMSD Calculations for Pol III S7-10

Figure S17. Normal mode analysis for Pol III with Pol-dA and Exo-Mut S11

Figure S18. Normal mode analysis for Pol III with DNA and Apo structure S12

Figure S19. Correlation analysis for Exo-mode wild type and mutant Pol III S13

Figure S20-21. Correlation analysis for Pol-mode wild type and mutant Pol III S14-15

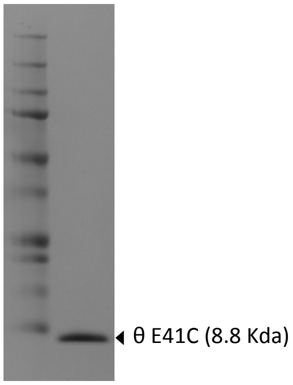

Figure S1. Polyacrylamide gel analysis of the purified θ E41C mutant protein.

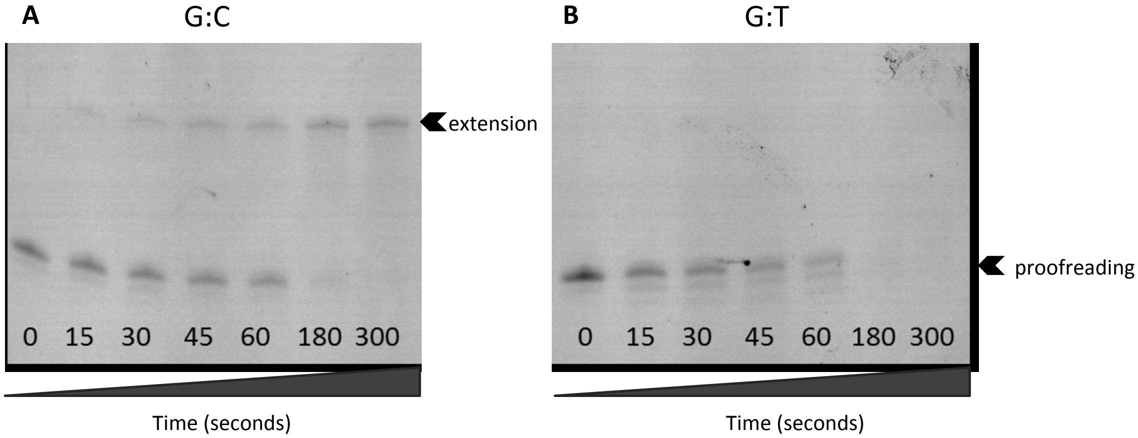

Figure S2. Time course primer extension and proofreading assays with wild type αβεθ to test the function of the Cy5 labeled protein. Primer extension and degradation assays were performed with matched (G:C) and mismatched DNA termini (G:T) (A) extension past a G:C terminus and (B) degradation at a terminal G:T mismatch. For DNA sequences, see Table 1 in the manuscript (note: in comparison to Table 1 the G:T DNA does not have a phosphorothioate linkage in order to observe Pol-mediated degradation).

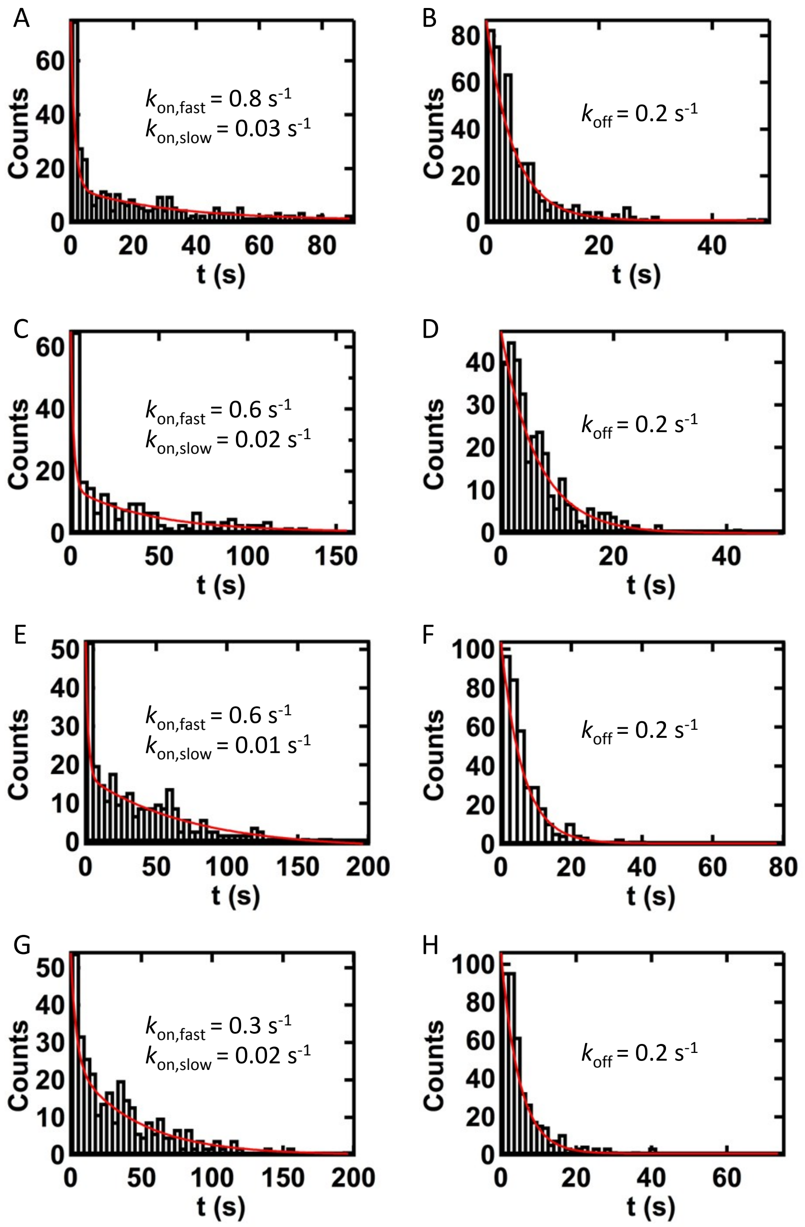

Figure S3. Binding exponential decay curves for wild type Pol III fit to a double exponential for the fast and slow k_{on} rates and to a single exponential for the k_{off} rate (A-B) G:ddC DNA (N = 104) (C-D) G:C DNA (N = 94) (E-F) G:T DNA (N = 103) and (G-H) G:A DNA (N = 102).

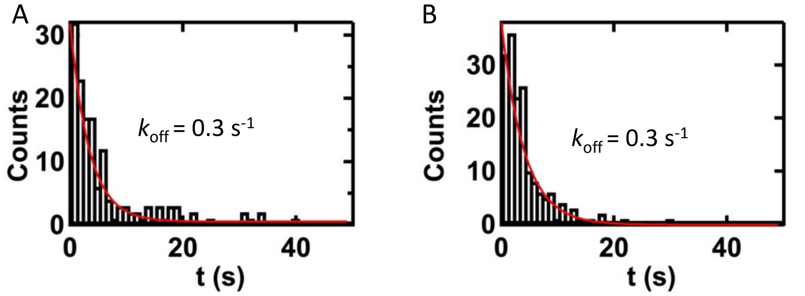

Figure S4. Binding exponential decay curves for Pol III at a double mismatch fit to single exponential for the k_{off} rate (A) wild type Pol III (N = 107) (B) mutant Pol III (N = 104).

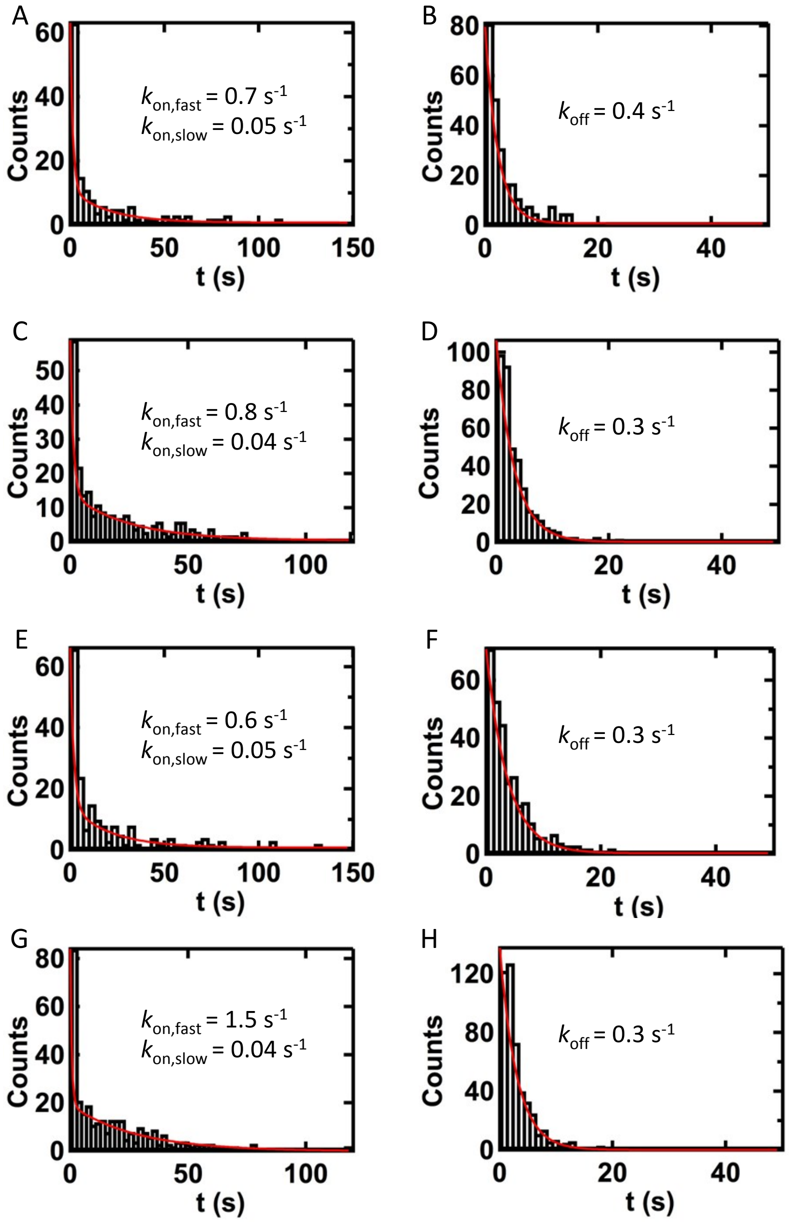

Figure S5. Binding exponential decay curves for mutant Pol III fit to a double exponential for the fast and slow k_{on} rates and to a single exponential for the k_{off} rate (A-B) G:ddC DNA (N = 99) (C-D) G:C DNA (N = 141) (E-F) G:T DNA (N = 104) and (G-H) G:A DNA (N = 143).

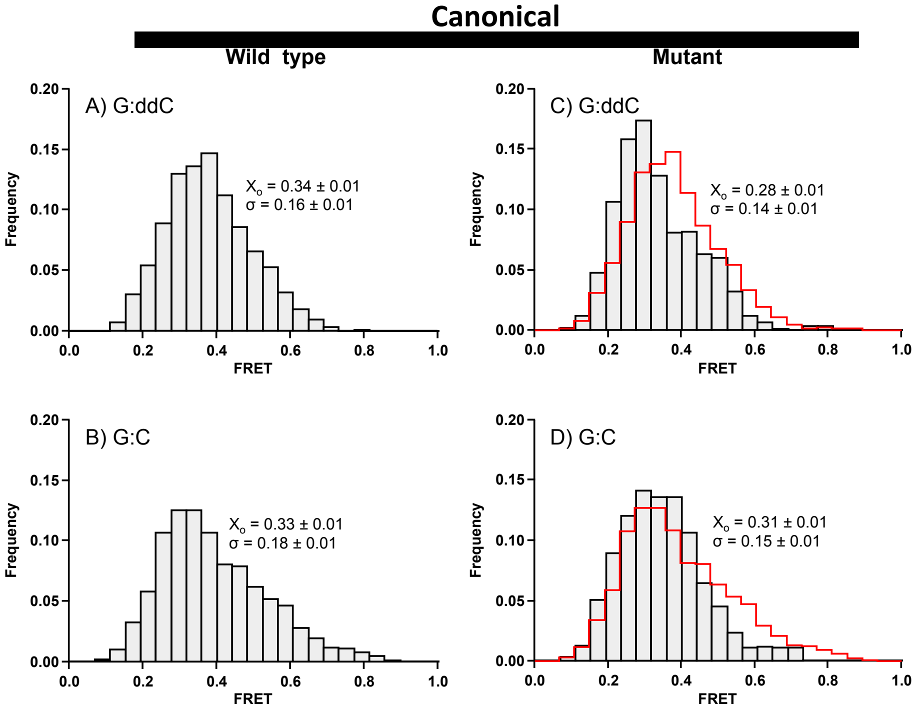

Figure S6. Distribution of dynamics observed for wild type and mutant Pol III as a function at canonical DNA paired termini (A, n = 105; B, n = 115; C, n = 206; D, n = 185).

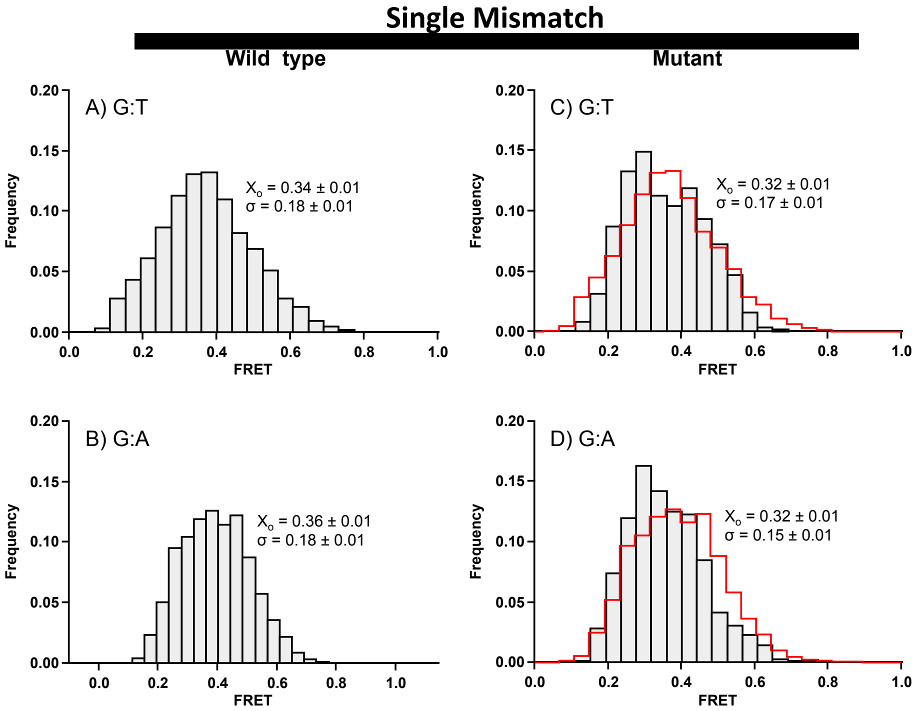

Figure S7. Distribution of dynamics observed for wild type and mutant Pol III as a function at a single DNA mismatch (A, n = 120; B, n = 111; C, n = 131; D, n = 114).

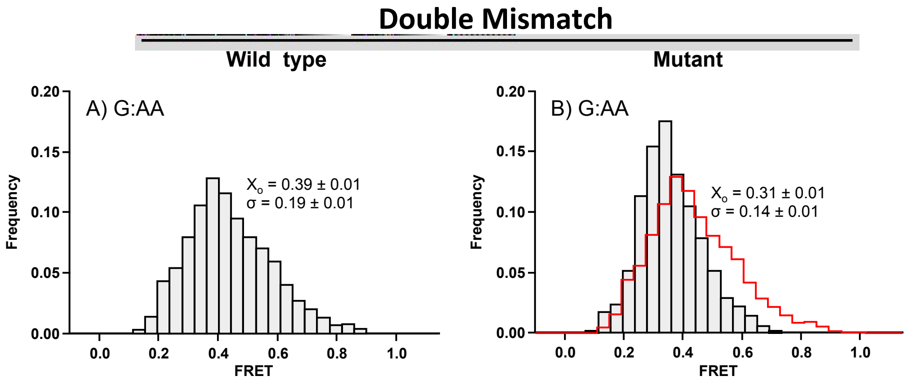

Figure S8. Distribution of dynamics observed for wild type and mutant Pol III as a function at a double DNA mismatch (A, n = 110; B, n = 103).

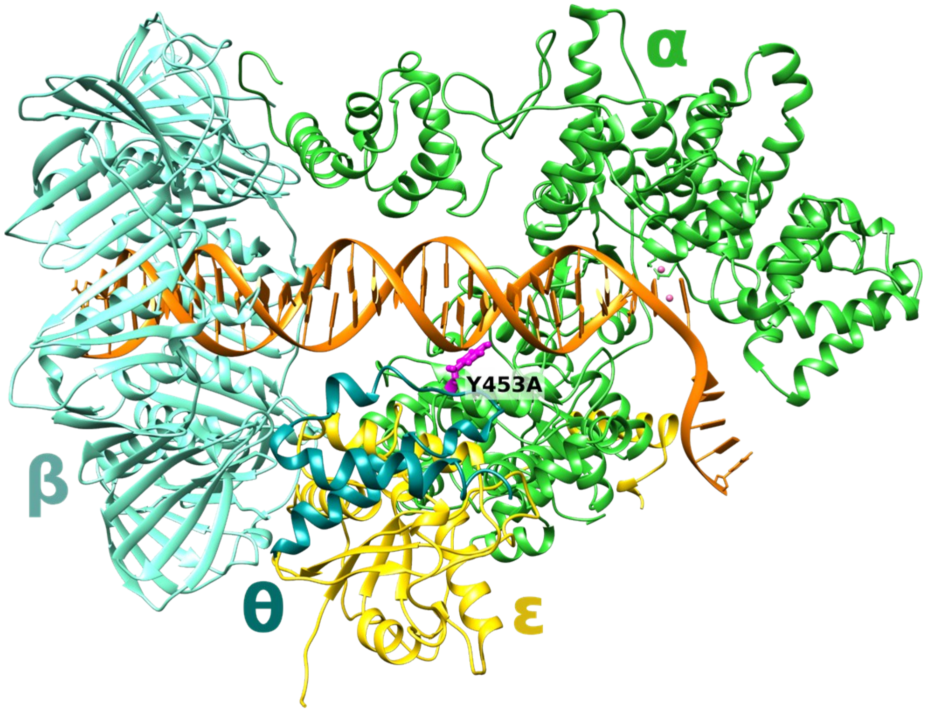

Figure S9. Schematic of overall structure of Pol III in the building mode, with the alpha domain colored green, the beta clamp domain colored light aqua, the epsilon proofreading domain colored in yellow, and the theta domain colored in dark cyan. The DNA is represented in orange, and the mutation site in hot pink (labeled Y453A).

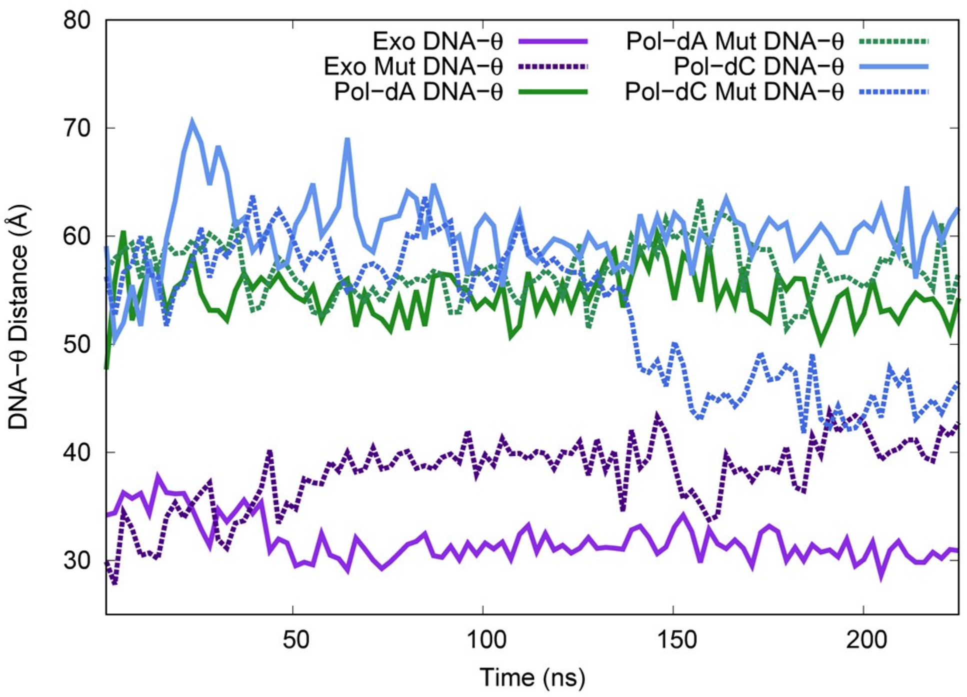

Figure S10. Calculated distances between DNA and theta subunit FRET tag locations over time for each MD trajectory, labeled by mode (Pol/Exo), correct (dC) or incorrect (dA) primer base at the primer/template terminus, and absence/presence of the Y453A mutation (Mut).

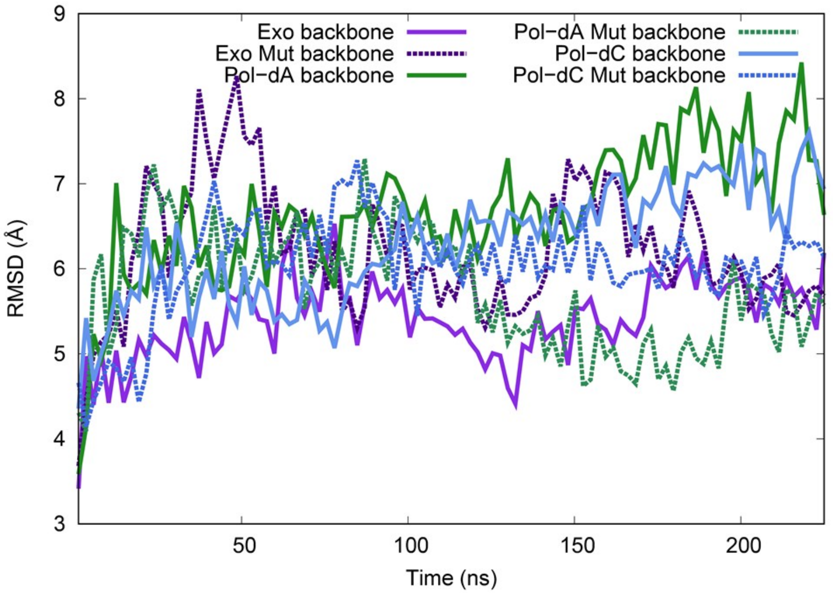

Figure S11. Overall RMSD for the backbone carbons of all subunits for each system, labeled by editing (Exo) or building (Pol) modes, correct (dC) or incorrect (dA) terminal primer base, and presence or absence of the Y453A mutation (Mut). Calculated relative to the cryo-EM structure used for the simulations, pdbid 5M1S for Exo and 5FKW for Pol.\

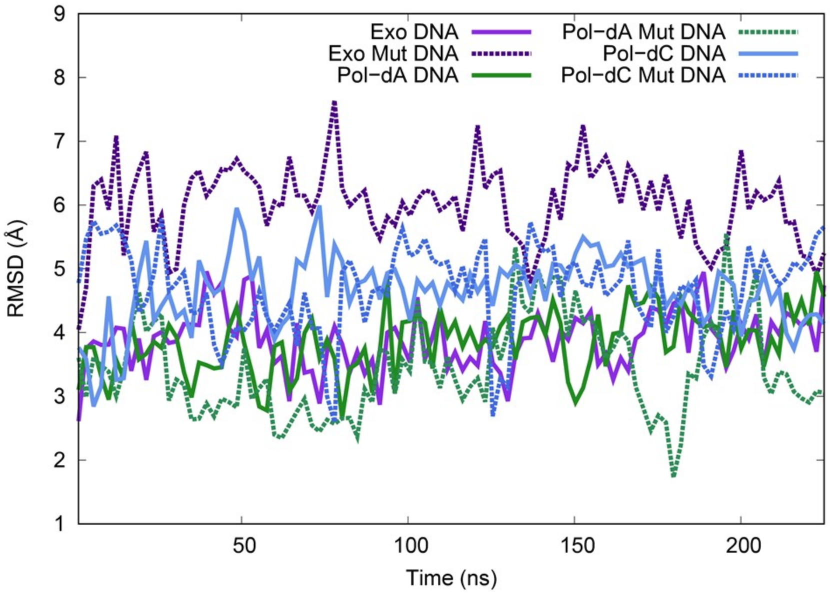

Figure S12. RMSD of DNA for each system, labeled by editing (Exo) or building (Pol) modes, correct (dC) or incorrect (dA) terminal primer base, and presence or absence of the Y453A mutation (Mut). Calculated relative to the cryo-EM structure used for the simulations, PDB codes 5M1S for Exo and 5FKW for Pol.

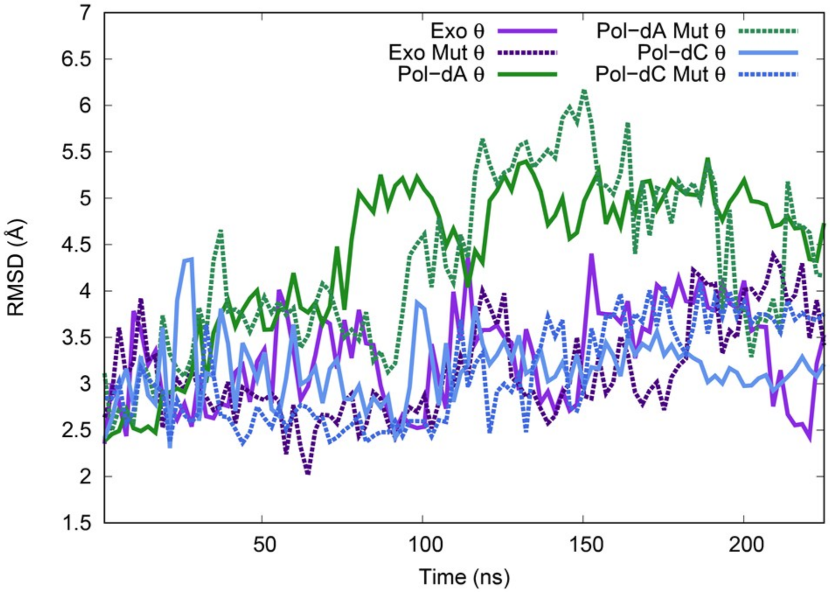

Figure S13. Backbone RMSD for the θ domain in editing (Exo) and polymerase (Pol) modes, correct (dC) or incorrect (dA) terminal primer base, and presence or absence of the Y453A mutation (Mut). Calculated relative to the cryo-EM structure used for the simulations, , pdbid 5M1S for Exo and 5FKW for Pol.

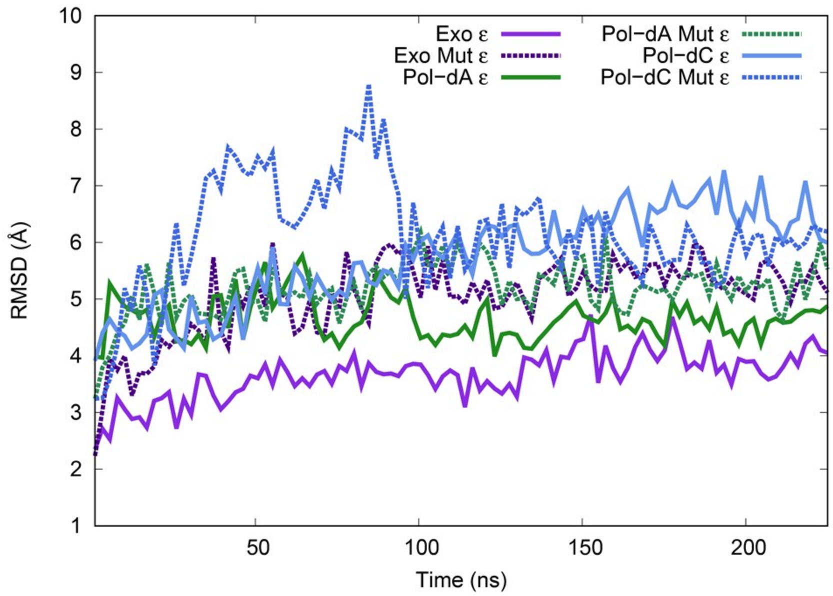

Figure S14. Backbone RMSD for the ε domain in editing (Exo) and polymerase (Pol) modes, correct (dC) or incorrect (dA) terminal primer base, and presence or absence of the Y453A mutation (Mut). Calculated relative to pdbid 2GUI.

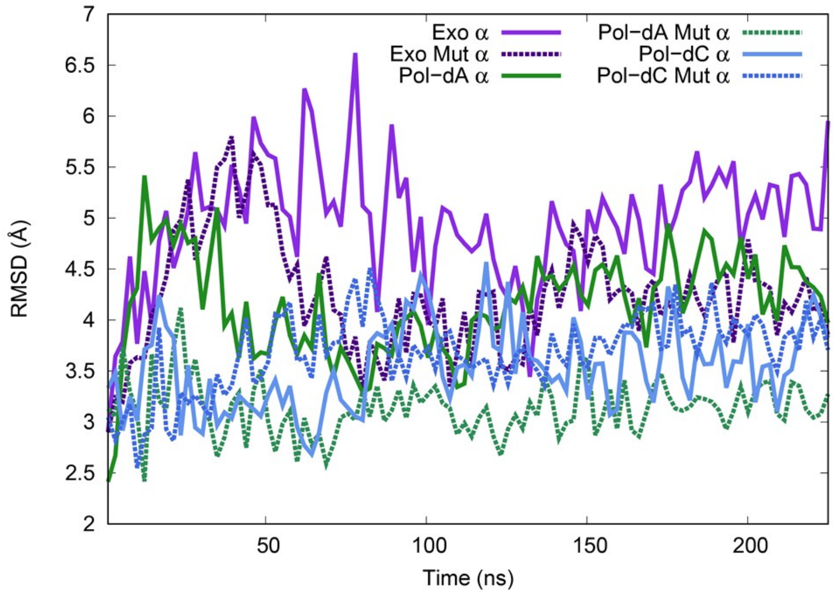

Figure S15. Backbone RMSD for the α domain in editing (Exo) and polymerase (Pol) modes, correct (dC) or incorrect (dA) terminal primer base, and presence or absence of the Y453A mutation (Mut). Calculated relative to pdbid 2HNH.

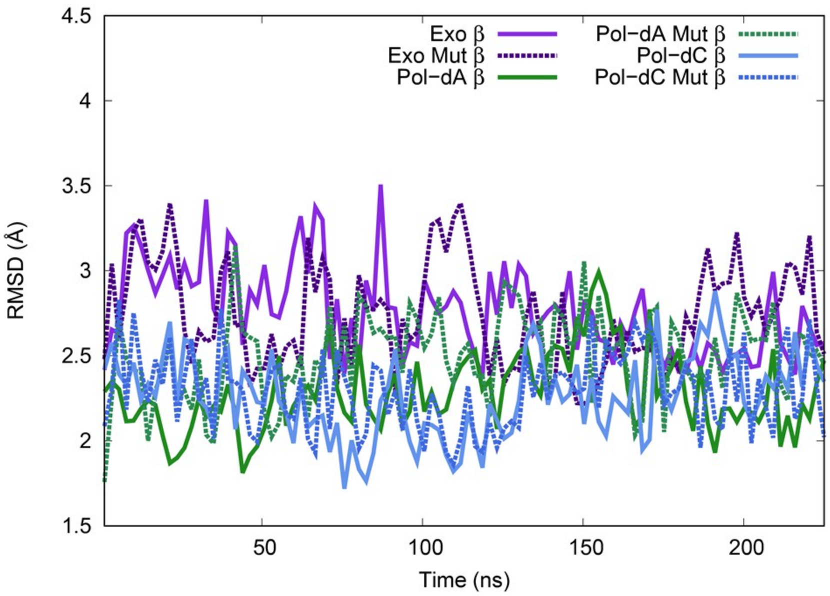

Figure S16. Backbone RMSD for the β domain in editing (Exo) and polymerase (Pol) modes, correct (dC) or incorrect (dA) terminal primer base, and presence or absence of the Y453A mutation (Mut). Calculated relative to pdbid 1MMI.

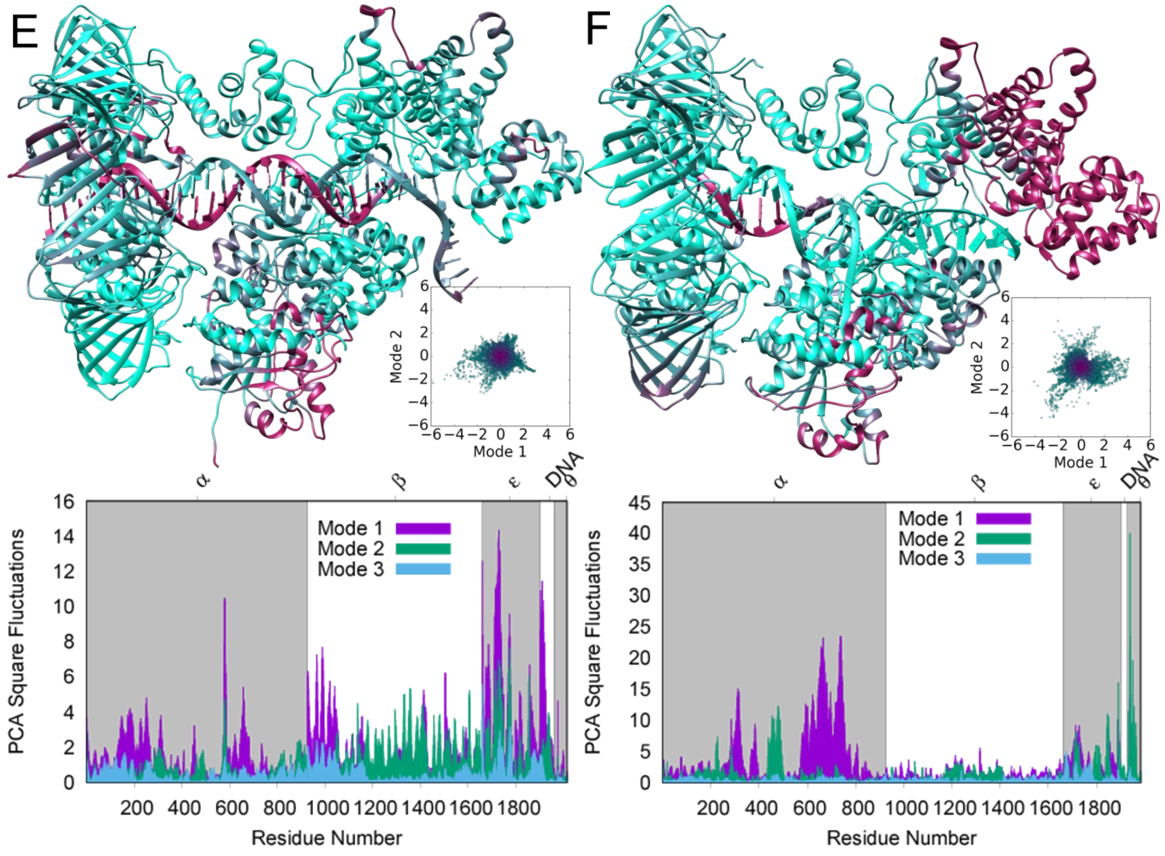

Figure S17. Square fluctuations for principle normal modes 1 and 2 (PCA) mapped on 3D protein structure and colored by relative amount of movement from most (mauve) to least (cyan), scatterplots of normal mode 1 versus normal mode 2 and per-residue square fluctuations for principle modes 1, 2 and 3 labeled by residue range for E. Polymerase mode with incorrect terminal primer base at the primer/template terminus (Pol-dA) and the Y453A mutation, F. Editing mode with the Y453A mutation (Exo-Mut).

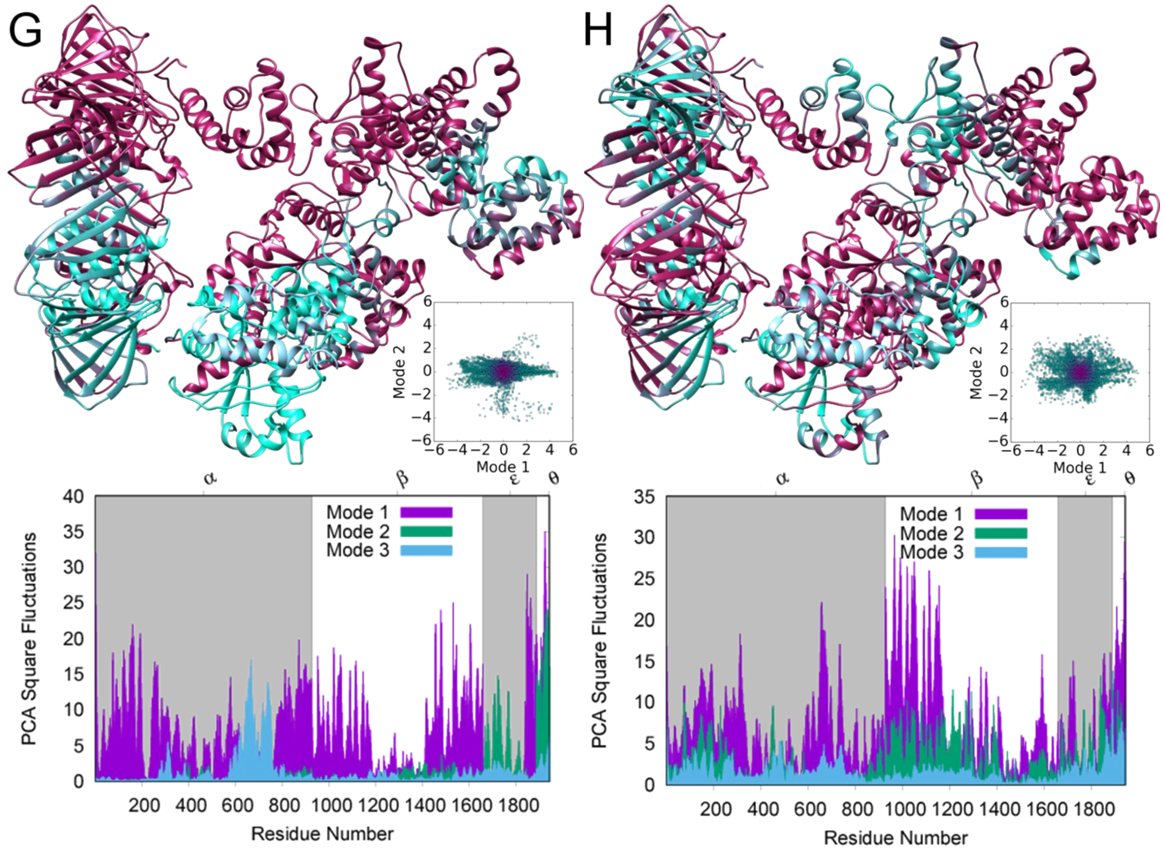

Figure S18. Square fluctuations for principle normal modes 1 and 2 (PCA) mapped on 3D protein structure and colored by relative amount of movement from most (mauve) to least (cyan), scatterplots of normal mode 1 versus normal mode 2 and per-residue square fluctuations for principle modes 1, 2 and 3 labeled by residue range for G. Apo mode, and H. Apo mode with the Y453A mutation.

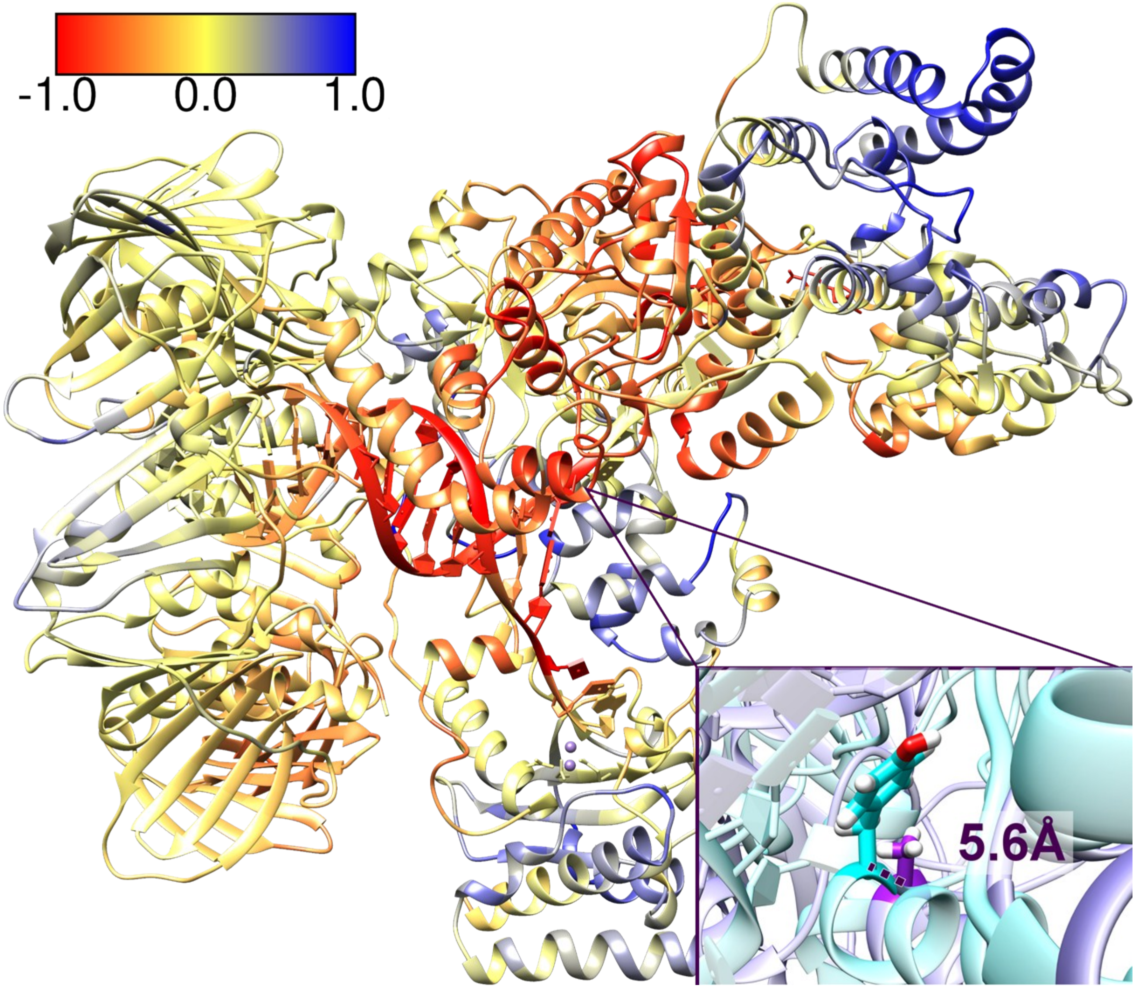

Figure S19. Difference correlation analysis between the Exo and Exo mutant structures extracted for residue and mapped onto the protein-DNA complex. Red denotes an increase in relative anticorrelation, blue an increase in relative correlation, and yellow little to no difference between Exo and Exo mutant. A close-up of the mutation location shows the mutant alanine in purple and the original tyrosine in cyan, with the final distance between their Cα denoted in dark purple.

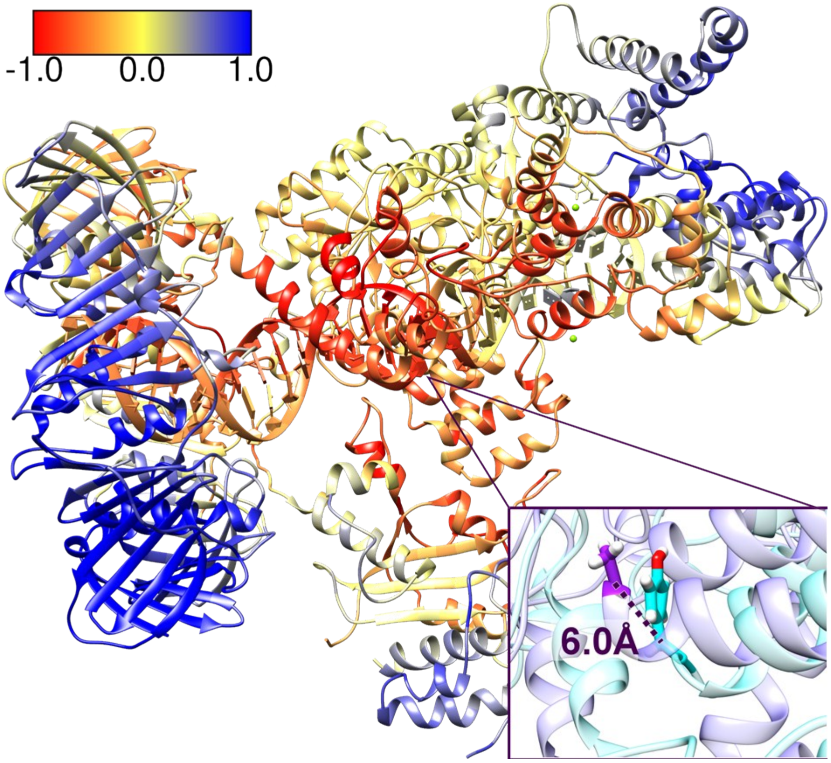

Figure S20. Difference correlation analysis between the Pol-dA and Pol-dA mutant simulations extracted for residue 453 and mapped onto the protein-DNA complex. Red denotes an increase in relative anticorrelation, blue an increase in relative correlation, and yellow little to no difference between Pol-dA and Pol-dA mutant. A close-up of the mutation location shows the mutant alanine in purple and the original tyrosine in cyan, with the final distance between their Cα denoted in dark purple.

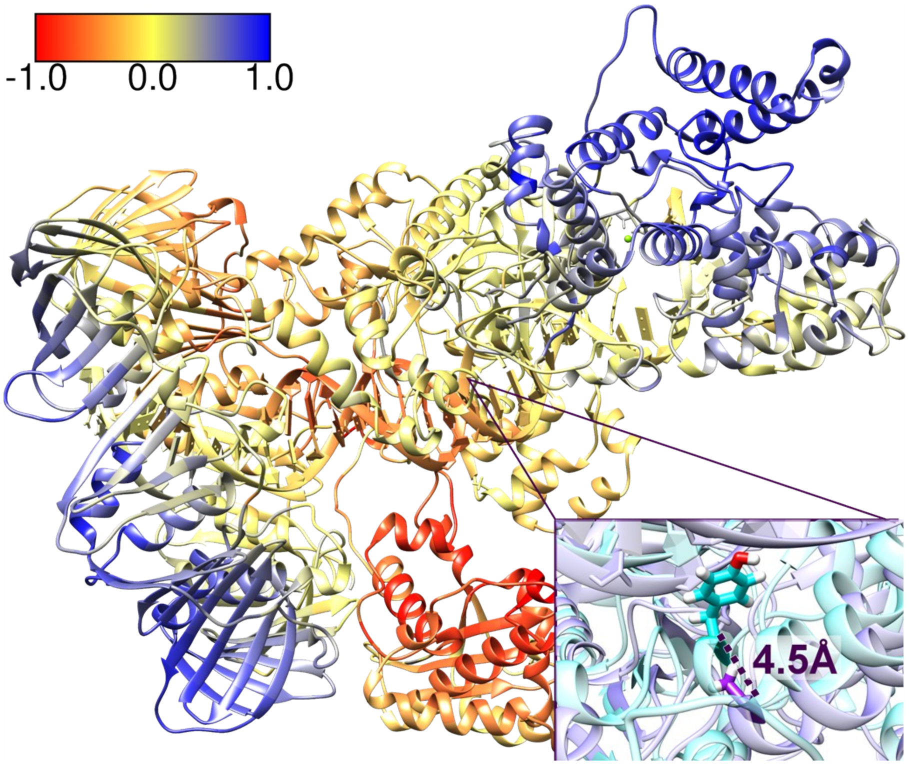

Figure S21. Difference correlation analysis between the Pol-dC and Pol-dC mutant structures extracted for residue 453 and mapped onto the protein-DNA complex. Red denotes an increase in relative anticorrelation, blue an increase in relative correlation, and yellow little to no difference between Pol-dC and Pol-dC mutant. A close-up of the mutation location shows the mutant alanine in purple and the original tyrosine in cyan, with the final distance between their Cα denoted in dark purple.
